# Site selective reading of epigenetic markers by a dual-mode synthetic receptor array†
†Electronic supplementary information (ESI) available: New molecule synthesis and characterization and additional spectral data. See DOI: 10.1039/c7sc00865a



**DOI:** 10.1039/c7sc00865a

**Published:** 2017-03-22

**Authors:** Yang Liu, Lizeth Perez, Magi Mettry, Adam D. Gill, Samantha R. Byers, Connor J. Easley, Christopher J. Bardeen, Wenwan Zhong, Richard J. Hooley

**Affiliations:** a Department of Chemistry , University of California – Riverside , Riverside , CA 92521 , USA . Email: wenwan.zhong@ucr.edu ; Email: richard.hooley@ucr.edu; b Environmental Toxicology Program , University of California – Riverside , Riverside , CA 92521 , USA; c Department of Biochemistry and Molecular Biology , University of California – Riverside , Riverside , CA 92521 , USA

## Abstract

Variably functionalized self-folding deep cavitands form an arrayed, fluorescent indicator displacement assay system for the detection of post-translationally modified (PTM) histone peptides. The hosts bind trimethyllysine (KMe_3_) groups, and use secondary upper rim interactions to provide more sensitive discrimination between targets with identical KMe_3_ binding handles. The sensor array uses multiple different recognition modes to distinguish between miniscule differences in target, such as identical lysine modifications at different sites of histone peptides. In addition, the sensor is affected by global changes in structure, so it is capable of discriminating between identical PTMs, at identical positions on amino acid fragments that vary only in peptide backbone length, and can be applied to detect non-methylation modifications such as acetylation and phosphorylations located multiple residues away from the targeted binding site. The synergistic application of multiple variables allows dual-mode deep cavitands to approach levels of recognition selectivity usually only seen with antibodies.

## Abstract 2

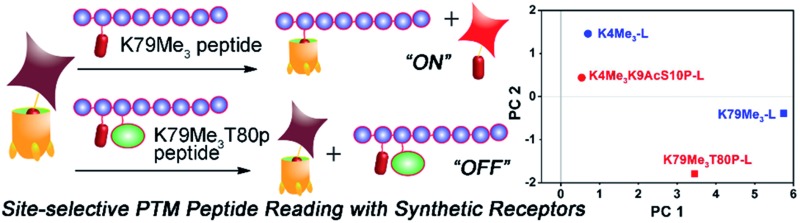
Arrayed, self-folding deep cavitands form a fluorescence displacement assay system for the site-selective sensing of post-translationally modified (PTM) histone peptides.

## Introduction

The diversity of proteins in living cells is greatly increased by post-translational modifications (PTMs).^1^ These PTMs change protein structure, leading to substantial effects on enzyme activity, protein–protein interaction and protein subcellular localization, impacting almost all dynamic cellular processes. Although great effort has been invested to identify PTMs in the proteome, it remains challenging due to their low abundance, highly dynamic modification states, and large variety in modification type and location.^2^ A major challenge is the discrimination between similar PTMs, especially positional variations in a single type of modification, and the detection of different modifications in a single target. Synthetic receptors provide an inviting solution to this problem, as they are cheap, easily synthesized, and can show selectivity for binding different residues on protein scaffolds.^3^

The major limitation in molecular recognition of biological targets using synthetic receptors is the lack of selectivity when compared to natural systems, especially antibodies.^4^ Whereas small molecule hosts must be synthesized from the ground up, natural systems can employ highly evolved superstructures and complex synthetic machinery to access receptors that show exquisite selectivity to small changes in environment.^5^ In contrast, small molecule hosts are generally based on stable, easily accessible macrocyclic systems that function in water, such as cyclodextrins,^6^ cyclophanes,^7^ cucurbit[*n*]urils,^8^ and calixarenes.^9^ These simple cavity-based species can recognize hydrophobic small molecules,^6*a*^ peptide fragments^7*a*^ and in some cases, even intact proteins.^3^ Some hosts have been applied toward the recognition and analysis of PTM proteins and peptides,^10^ and employed in arrays for differential analysis.^9*c*^ The most widely applied and successful small molecule hosts for biomacromolecules are tetrasulfonatocalixarene (CX4) and cucurbit[7]uril, (CB7). While the parent macrocycles have impressive recognition capabilities, synthetic variations are extremely difficult.^11^ Other hosts can be more easily varied, but their effectiveness is far more limited. The true difference between antibodies and small molecule receptors is the ability to recognize not only the residue of direct interaction, but to be able to discriminate based on adjacent residues and the surrounding environment. Antibodies are specific to residue location, not just residue type, whereas synthetic receptors show pan-specificity for the encapsulated functional group. Discrimination between highly similar PTMs, dual modifications of the same type, or positional variations between identical PTMs is extremely challenging, as most synthetic receptors often only have one recognition component: that of the PTM group itself (*e.g.* phosphate).^12^ Most receptors are not sufficiently selective to allow discrimination between identical PTMs in different environments, as that requires selective secondary interactions.

The solution to this problem is to employ not one receptor, but multiple, variably functionalized receptors at once. An array formed from multiple receptors can provide multi-mode recognition to maximize signal differences from small variations in guest molecules, enabling more selective target discrimination. This technique has been used to create a “chemical nose” for small molecules in a variety of optical sensing applications.^13^ Targets such as glycoproteins,^14^ phosphorylated peptides^15^ and sugars^16^ can be discriminated using a functional group sensitive chemical sensors.^17^ The combined responses from selective interaction between many individual receptors and analytes generate a distinct pattern (fingerprint) for each analyte that can be analysed using a variety of multivariate statistic tools such as principal component analysis (PCA)^18^ or linear discriminant analysis (LDA).^19^ A pioneering example of this concept was shown by the Hof group,^9*c*^ which used a lucigenin:CX4 indicator displacement assay to read methylation PTMs in histone peptide fragments, based on selective recognition of methylated arginine and lysine residues. The challenge in further application of this system is the lack of variability of the CX4 receptors. For maximal target discrimination, multiple variables in host binding motif are required. Dual, orthogonal recognition motifs in a single receptor scaffold could achieve more complex target discrimination *via* pairing an “anchor” recognition motif with secondary effects. The most obvious strategy is to pair shape-based recognition (*via* a synthetic cavity) with H-bonding and charge matching. Dual-mode binding is a well-established phenomenon in supramolecular chemistry, whereby a cavity-based host is combined with a second recognition element that allows further discrimination.^20^ Cucurbit[*n*]urils are the best example of this: some extremely high affinities can be observed with suitably sized alkylammonium species, based on a combination of properly oriented hydrogen bonds and hydrophobic interactions.^21^ The lack of tunability of CBs limits their use in a multi-mode binding array, however. The best combination of tunability and target affinity in aqueous supramolecular hosts lies with deep, self-folding cavitands. Here we show that a suite of upper rim functionalized self-folding deep cavitands can be applied as fluorescent displacement sensors in an array-based format, and show exquisite selectivity in discriminating between highly similar small molecule targets and positional variations in histone peptides carrying lysine methylation, phosphorylation and acetylation PTMs. The discrimination occurs *via* multiple different recognition/displacement phenomena, rather than a simple cavity-based recognition process.

## Results and discussion

**Fig. 1:**
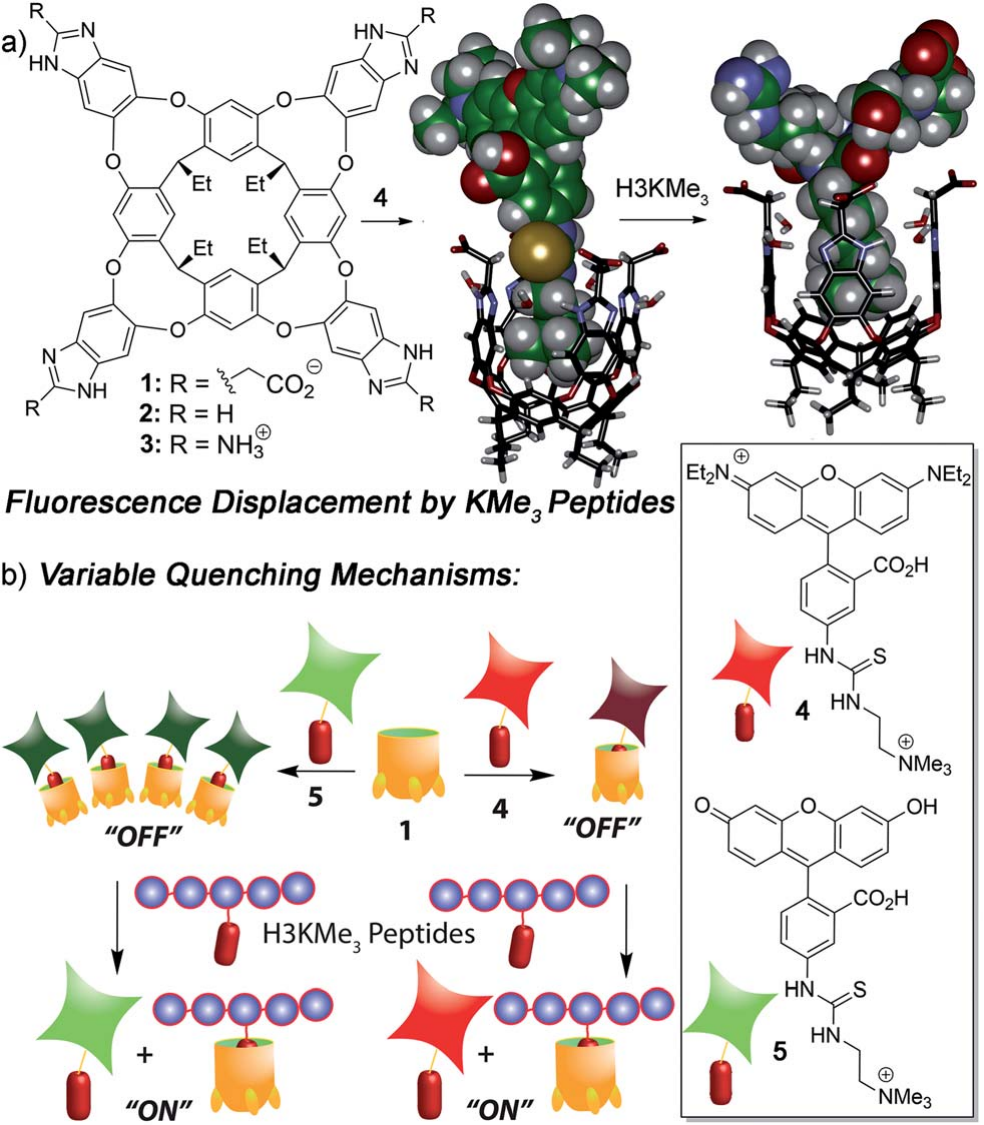
(a) Structure of hosts **1–3** and minimized models of the **1·4** and the **1**·(AR(KMe_3_)ST) host:guest complexes (SPARTAN); (b) illustration of the FDA processes and structure of rhodamine B guest **4** and fluorescein guest **5**.

We focused on self-folding deep cavitands based on benzimidazole scaffolds, as these hosts are water-soluble and can be easily varied at the upper rim, introducing groups of varying size, hydrophobicity and charge, while keeping the target binding cavity constant. Cavitands such as **1** are flexible,^22^ but are held in a kinetically stable “vase-like” conformation (Fig. 1a) in the presence of water. This host shows good selectivity for soft R-NMe_3_^+^ cations such as choline or trimethyllysine.^23^ Choline has a *K*_d_ of ∼50 μM in pure D_2_O solution, driven by favourable cation–π interactions with electron rich walls: analogous targets such as dimethylammonium salts or NEt_4_^+^ ions are poor shape matches for the cavity. These hosts are well-suited for recognizing lysine trimethylation PTMs, as the trimethylammonium group in KMe_3_ fits well in the cavitand, and other methylations (KMe_0_, KMe_1_ and KMe_2_) show much weaker affinity. In this study, we employed three specific cavitands (Fig. 1a): anionic **1**,^23*b*^ neutral **2** ^24^ and cationic **3**.^25^ While the cavity size is identical in each host, the upper rim functions vary in size, charge, hydrophobicity and H-bonding capability.

We have previously shown that the tetracarboxylate deep cavitand **1** is capable of recognizing lysine trimethylation PTMs on histone H3 peptide fragments *via* selective fluorescence displacement.^26^ The binding of fluorescein guest **5** in host **1** causes aggregation of the lipid-like host (Fig. 1b) and concomitant aggregation-based quenching of the guest. Fluorescence recovery occurs upon displacement of **5** by the desired KMe_3_ target.^26^ While this sensing system was effective for discriminating between histone KMe_3_ and KMe_0/1/2_, the more challenging task of site-selective discrimination requires more variables, and so we synthesized the simple rhodamine B variant, guest **4**. Guest **4** was synthesized from nitro-rhodamine B in two steps (see ESI† for procedures and characterization). Surprisingly, despite its similarities to guest **5**, RhB guest **4** showed remarkable differences in binding behaviour: the affinity for cavitands **1–3** is stronger, and the quenching does not rely on an aggregative mechanism, but occurs upon simple 1 : 1 complex formation.

**Fig. 2:**
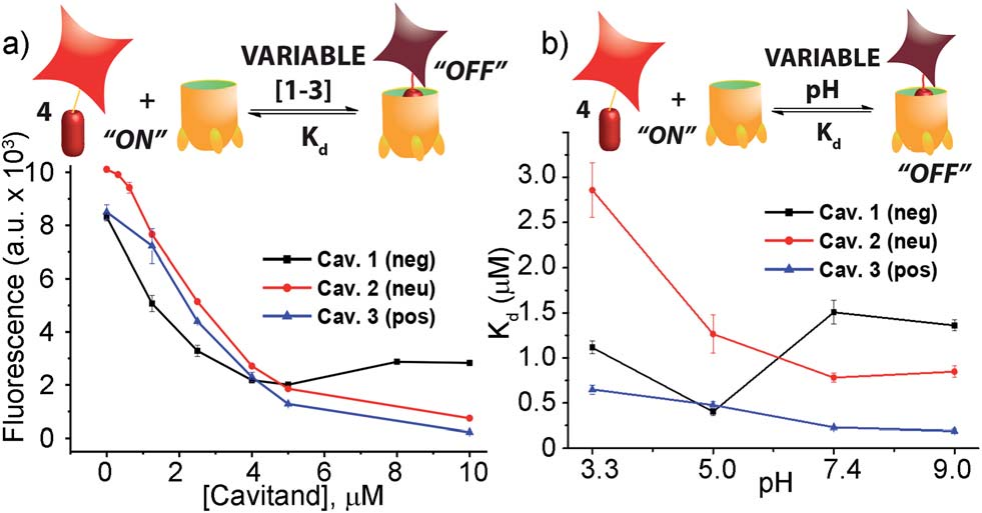
(a) Fluorescence quenching of 3 μM guest **4** with varying cavitand [**1–3**] in 80 mM phosphate buffer, pH = 7.4; (b) pH-dependent affinity of guest **4** for cavitands **1–3** in 80 mM different buffers (citrate buffer, pH = 3.3; phosphate buffer, pH = 5.0; phosphate buffer, pH = 7.4; carbonate buffer, pH = 9.0).

When guest **4** (3 μM) was mixed with cavitand **1**, **2** or **3** in phosphate buffer, a strong loss in fluorescence was observed in each case. Fluorescence reached a minimum at [**1**] = 4 μM, with 20% of the original fluorescence retained (Fig. 2a). Cavitands **2** and **3** showed stronger quenching than **1**: the guest fluorescence continued to drop to only 7% and 2% of the original value with increasing [cavitand] up to 10 μM for **2** and **3**, respectively.

Fluorescence life-time measurements supported a static quenching mechanism for the interaction between guest **4** and all three cavitands (see Fig. S-19†). The Stern–Volmer equation was therefore used to calculate the dissociation constants (*K*_d_) for the host–guest pairs, using a 1 : 1 binary complex formation model. Since the protonation state for all three cavitands and guest **4** varies with pH, we measured the fluorescence quenching curves at various pH values, pH 3.3, 5.0, 7.4, and 9.0, and evaluated the dependence of *K*_d_ on pH. As shown in Fig. 2b and Table 1, RhB guest **4** shows extremely strong affinities for all three hosts **1–3**, with *K*_d_ values in the micro- and sub-micromolar range. Cavitands **2** and **3** both show increasing affinity for **4** at increasing pH, but tetracarboxylate host **1** shows greatest affinity at pH 5.0. The weakest affinity is seen for **2·4** at pH 3.3, with *K*_d_ = 2.86 μM, and the strongest affinity is between **4** and cationic **3** at pH 9.0. In that case, the dissociation constant is 190 nM, which is an affinity usually only seen between suitable guests and CB[*n*],^11,21b^ rather than flexible cavitand hosts.

**Table 1:** Dissociation constants (*K*_d_, μM) for guest **4** in hosts **1–3** in varying pH conditions

Host	pH 3.3	pH 5.0	pH 7.4	pH 9.0
1	1.11 ± 0.07	0.40 ± 0.04	1.51 ± 0.13	1.36 ± 0.06
2	2.86 ± 0.30	1.27 ± 0.21	0.78 ± 0.05	0.85 ± 0.06
3	0.65 ± 0.05	0.48 ± 0.04	0.23 ± 0.04	0.19 ± 0.02

These strong affinities are extremely encouraging for the application of the RhB guest–cavitand pairs in fluorescence displacement assays of biorelevant target binding. The high degree of quenching reduces the background signal in the absence of displacement, and only strongly bound guests are capable of displacing **4**, inducing large signal change, reducing “false positive” hits. Importantly, guest **4** does not show any variation in emission efficiency at varying pH (see Fig. S-20†). The constant native emission of displaced **4** in various media enables the use of different binding media as array elements, to provide selective, pH responsive guest recognition. The fact that the displacement can occur in aqueous buffered solutions makes the assay simple and highly biocompatible.

**Fig. 3:**
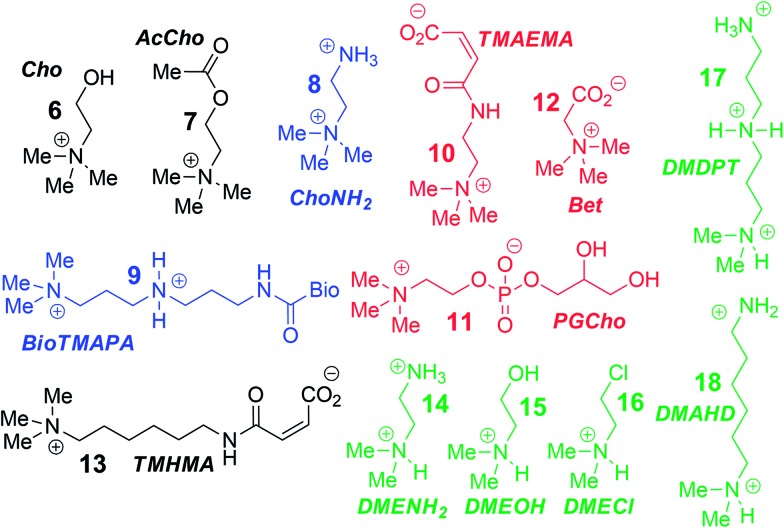
Small molecule guest scope.

To analyse the scope of the array, and to determine how effective the array is at discriminating extremely small differences in target, we focused on a suite of synthetic small molecule targets. The array was constructed by incorporating the three cavitands (**1–3**) and their complexes with the two fluorescent guests (**4** and **5**) at different pHs (pH 3.3, 5.0, 7.4, and 9.0 for guest **4**; and pH 7.4 and 9.0 for guest **5**), with a total of 14 variables. The concentrations of cavitand and fluorescent guest were maintained at 4 and 3 μM, respectively. Variable pH was achieved by addition of 70 mM of the sodium citrate (pH 3.3), phosphate (pH = 5.0 and 7.4), and carbonate (pH 9.0). Fig. 3 shows the initial small molecule targets for testing: these have large differences in some cases, with a series of NMe_3_^+^ (**6–13**) and NHMe_2_^+^ (**14–18**) anchors.

More subtle variations are also included in the two substrate pools, with the variations positioned such that they interact closely with the upper rims of cavitands **1–3**. These include cationic ammonium groups varying in position and p*K*_b_ (**8**, **9**), neutral, yet H-bonding groups (**6**, **7**), anionic guests (**10–12**), and guests with lipophilic chains (**13**). The NMe_2_H^+^ guests **14–18** mirror these variations as well. The guests were mixed with each sensor solution in 96-well plates at a fixed concentration of 100 μM, to ensure detectable displacement signal, even for very weak competitors. The fluorescence was measured in a plate reader before (*F*_min_) and after mixing (*F*) and compared with the original fluorescence of **4** and **5** (*F*_max_), to determine the % of guest displaced (*F* – *F*_min_)/*F*_max_. Comparison of these percentages attained allows analysis of the relative affinities of the target guest screen, with greater displacement indicating higher target affinity. Even though **4** and **5** are much better guests than the small molecule targets, sufficient displacement occurs, allowing mechanistic analysis of the recognition process.

**Fig. 4:**
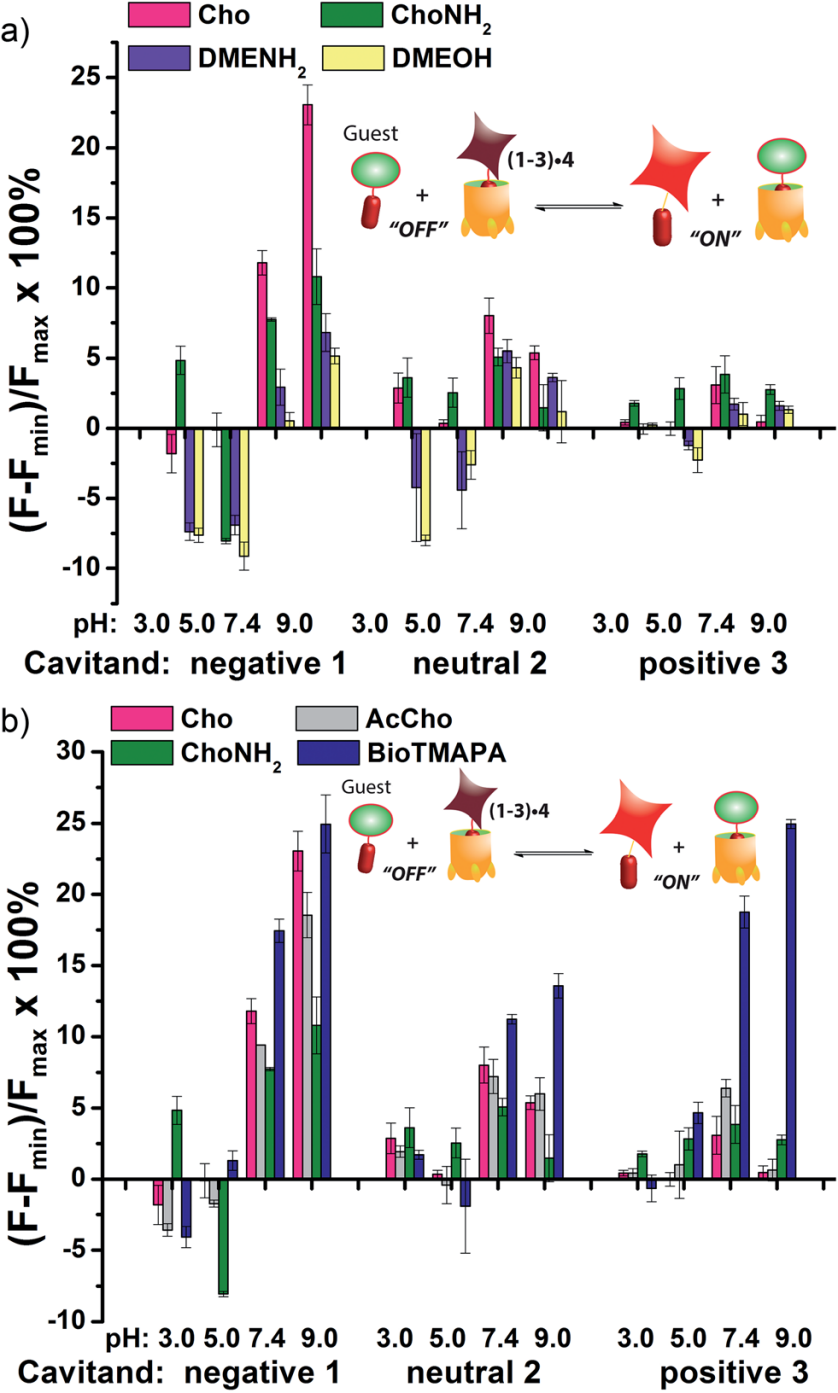
Small molecule indicator displacement. (a) R–NMe_3_^+^ *vs.* RNMe_2_H^+^ discrimination for guests **6**, **8**, **14**, **16**; (b) discrimination between highly similar R–NMe_3_^+^ guests **6–9**. Error bars calculated from three repeat experiments. For negative **1**, [guest **4**] = 3 μM and [cavitand **1**] = 4 μM, and for neutral **2** and positive **3**, [guest **4**] = 3 μM, [cavitand **2** or **3**], = 5 μM. [small molecule] = 100 μM.

The screening data shown in Fig. 4 and 6 (as well as Fig. S-21†) illustrate the sensitivity of the system to extremely small differences in guest properties. Fig. 4a shows the simplest discrimination between two R–NMe_3_^+^ guests (choline **6** and cholamine **8**), and two less favoured R–NMe_2_H^+^ guests (*N*,*N*-dimethylethylenediamine **14** and *N*,*N*-dimethylethanolamine **15**), and provides a stark illustration of the effect of pH and cavitand type on four extremely similar guests. As expected,^23*b*^ the maximal displacement of guest **4** from cavitand **1** occurs with the two R–NMe_3_^+^ guests, which show >12% and ∼8% increase upon addition of **6** and **8** respectively at pH 7.4. More displacement (22%) is seen at pH 9.0 for guest **6**. For cavitands **2** and **3**, which display stronger affinities to guest **4**, a lower overall percent displacement is obtained. Despite this, R–NMe_3_^+^ guests **6** and **8** gave greater displacement than the R–NMe_2_H^+^ guests **14** and **15** in general, which only displaced less than 5% of **4** at basic pH conditions and negligible or even negative changes at acidic pH conditions for all three hosts. This is consistent with their lower affinity for **1–3**, illustrating that discrimination between these large variations is simple.

Fig. 4b shows the application of the array to more challenging discrimination between highly similar targets, namely four different R–NMe_3_^+^ guests: **6** and **8**, as well as acetylcholine **7** and ammonium BioTMAPA guest **9**. Variation of only a single group from OH (in **6**) to NH_2_ (in **8**) leads to noticeable pH-dependent differences in binding, especially to cavitand **1**, even though the binding anchors are identical. In acidic media (both pH 3.3 and 5.0), minimal displacement of **4** was observed for all guests **6–9**. This could be partially attributed to the higher affinity of the fluorescent guest **4** to cavitand **1** at acidic pH than at higher pHs (see Fig. 2b). At neutral and basic pH, much higher percentages of fluorophore **4** than that observed under the acidic conditions were displaced by **6–9** from all three hosts, with cavitand **1** showing the largest changes. The most interesting observation is that BioTMAPA **9** shows a larger displacement effect than choline **6** and acetylcholine **7** for **2** and **3**, even though the upper rims are ostensibly charge “mismatched”.

**Fig. 5:**
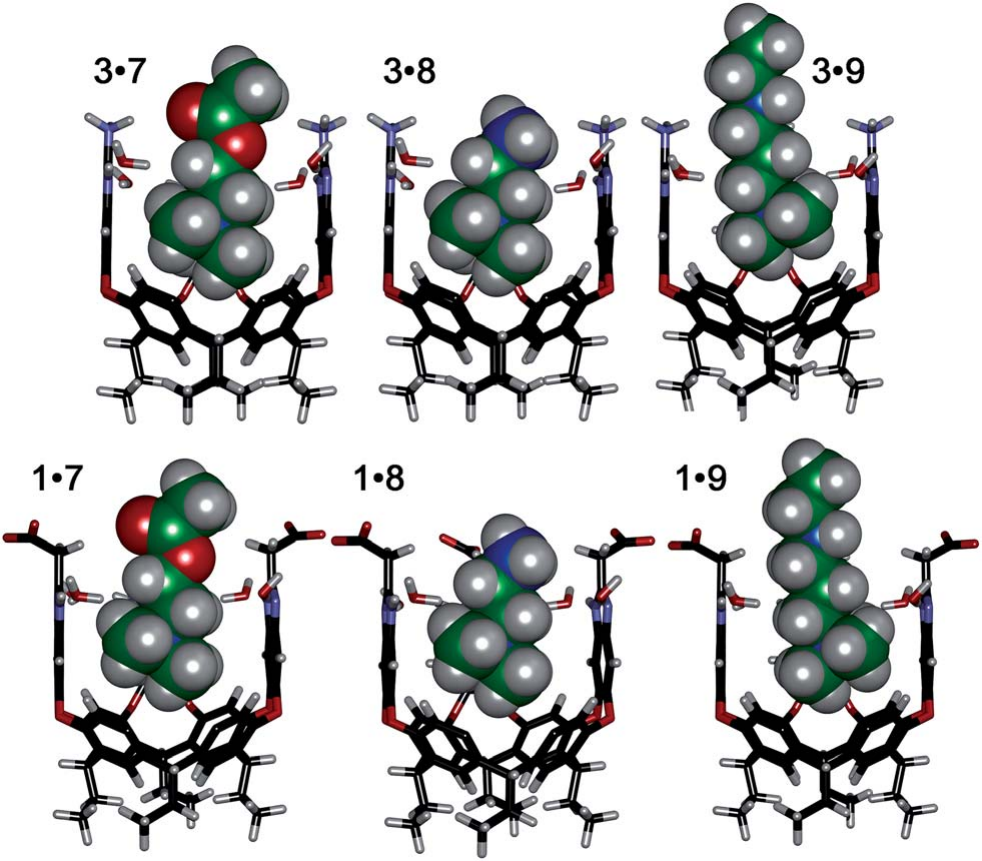
Minimized structures of the complexes between cavitands **1** and **3** (front walls removed for clarity), and guests **7**, **8** and **9** (structure of **9** shortened for clarity), SPAR TAN, AM1 forcefield.

The interplay between solvation, charge matching effects and H-bonding between the four guests **6–9** and cavitands **1–3** is complex, and the individual effects on affinity are challenging to extract. The increase in host capabilities for neutral guests in neutral **2** at basic pH has been previously described as due to a “tightened” hydrogen bonding seam that shrinks the cavity size and increases non-covalent space-filling and CH–π interactions with bound guest.^27^ However, both **1** and **3** contain upper rim groups that can also display variable protonation states, as can guests **8** and **9**. Variations in protonation state of some or all of the CO_2_^–^/CO_2_H groups in **1**, or the NH_2_/NH_3_^+^ groups in **3** in differing pH conditions, as well as variations in guest protonation state will affect the affinity. Fig. 5 shows minimized structures of the complexes formed between hosts **1** and **3**, and guests **7–9**, and illustrates that these affinity variations are most likely due to upper rim charge matching, rather than variations in steric or shape-fitting effects. The NMe_3_^+^ group fits snugly in the binding pocket, positioning the OH, NH_3_^+^ and NH_2_R^+^ groups of **6**, **8** and **9** in proximity with the upper rim host functions. For example, the only difference in upper functionality between **8** and **9** is that the amine in **9** is more basic (calculated p*K*_a_ for **9** is 9.81, and for **8** is 7.88) and is positioned one atom higher in the cavitand upon binding than **8**. At pH 7.4 when both **8** and **9** are protonated, the ammonium group in **9** is better positioned for favourable H-bonding and charge matching with the anionic CO_2_^–^ groups in host **1**.

**Fig. 6:**
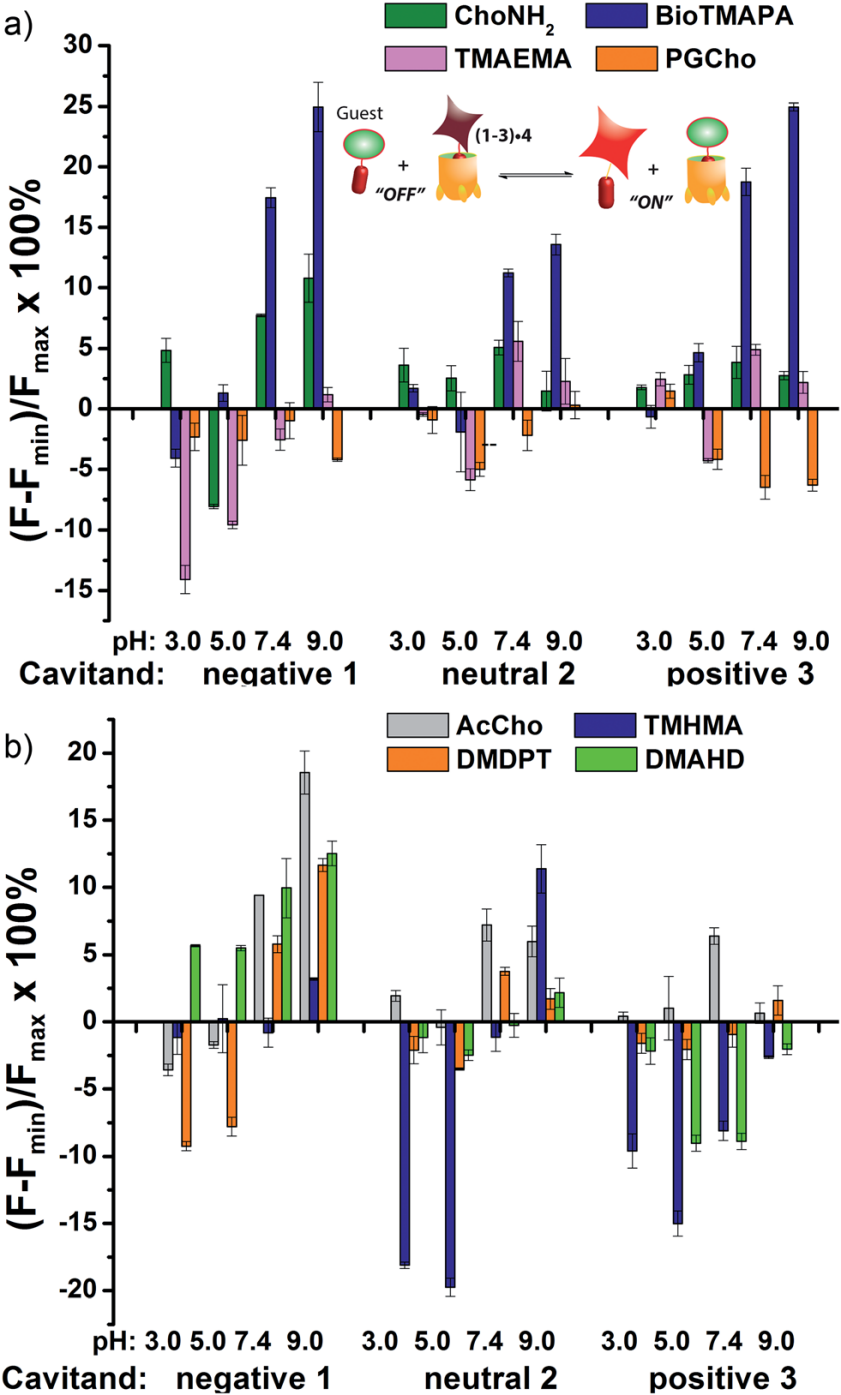
Small molecule indicator displacement. (a) Discrimination between anionic and cationic R–NMe_3_^+^ guests **8–11**; (b) discrimination between neutral and lipophilic guests **7**, **13**, **17**, **18**. Error bars calculated from three repeat experiments. For negative **1**, [guest **4**] = 3 μM and [cavitand **1**] = 4 μM, and for neutral **2** and positive **3**, [guest **4**] = 3 μM, [cavitand **2** or **3**], = 5 μM. [small molecule] = 100 μM.

The relatively large change in fluorescent signal upon displacement enabled the use of a competition assay for closer examination of the binding affinity of these compounds to cavitand **1**. By adding 0–5 mM of these small molecules to the mixture of **4** and **1** at fixed concentrations under these two pHs, fluorescence *vs.* [guest] titration curves were obtained. Using standard methods for determination of inhibitor binding constant in protein-ligand-inhibitor binding assays (see Experimental section), the dissociation constants (*K*_i_) of the complexes formed between cavitand **1** and guests **6–9** were calculated (see Table S-2† for a list of calculated *K*_i_ values). The *K*_i_ for **9** (*K*_i_ = 10.5 μM) is greater than that of **8** (*K*_i_ = 16.5 μM) at pH 7.4. At pH 9.0, cholamine **8** is completely deprotonated, causing a mismatch that lowers the affinity dramatically (*K*_i_ = >100 μM). The more basic BioTMAPA **9** is still partially protonated at this pH and while its affinity drops, it is only lowered to 69.6 μM. The affinities of the upper rim-neutral choline **6** (*K*_i_ = 9.9, 10.2 μM) and acetylcholine **7** (*K*_i_ = 13.9, 11.4 μM) for cavitand **1** are unaffected by charge mismatching, and remain similar at both pH 7.4 and 9.0. Larger structural variations in guest lead to more obviously explainable affinity variations, as shown in Fig. 6a. This series pairs guests with cationic groups at the upper rim (**8** and **9**) with guests displaying anionic groups (phosphatidyl-glycerol (PGCho) **11** and maleamate guest (TMAEMA) **10**). These effects are most pronounced for anionic host **1**. Whereas cationic guests **8** and **9** show strong affinity for **1**, these two anionic guests (**10** and **11**) positions its carboxyl or phosphate group directly at the carboxylates in **1** and shows no affinity at all under neutral and basic conditions for all three cavitands, suggesting that the phosphate or carboxyl group is also repelled by the electron-rich walls of the hosts.

As well as shape, charge and H-bond matching between host and guest, either in the cavity or at the upper rim, other effects can occur under select conditions, leading to unusual displacement results, as shown in Fig. 6b. Guests **13** (TMHMA) and **18** (DMAHD) are more lipophilic than their smaller counterparts, as they have *n*-hexyl groups in their interior structure. Addition of guest **13** at low pH causes a significant lowering of fluorescence, most notably with the complexes of **4** and cavitands **2** and **3**. This effect is also seen to a lesser extent with TMAEMA **10** with cavitand **1** (Fig. 6a). Competitive binding of guest process causes the fluorescence of **4** to recover upon displacement, so the presence of increased quenching upon guest addition was surprising. However, this phenomenon can be explained by aggregative effects. The extra fluorescence decrease only occurs for lipophilic R–NMe_3_^+^ or R–NMe_2_H^+^ guest at acidic pH, conditions that neutralize the carboxylate group in **13**, for example. Protonated **13** is structurally similar to dodecyl–NMe_3_^+^, which is a surfactant and forms micelles at millimolar concentrations. Evidently **13** also forms micelles, which incorporate the **2·4** complex, causing additional aggregation-based quenching of **4**.^26^ While unexpected, this phenomenon provides another variable for discrimination of lipophilic guests, one that is not dependent on cavity-based recognition.

**Fig. 7:**
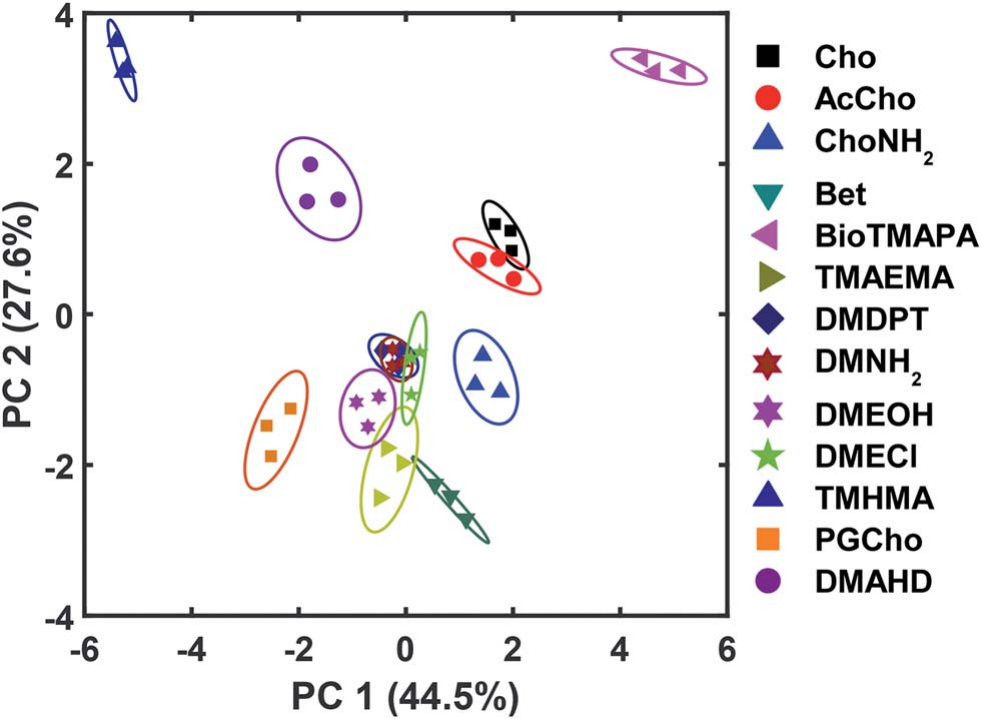
PCA plot for the small molecule screen. Each symbol represents one repeat of the measurement, and each molecule was tested 3 times, giving 3 identical symbols for each guest. The error ellipses were obtained at 95% confidence interval.

The wide variety of individual effects at play in this system, from the pH-dependent affinity of the fluorophores for the different cavitands, to guest matching and mismatching with the host upper rims and unexpected aggregation effects, is illustrated by Principal Component Analysis (PCA), as shown in Fig. 7. The scores plot was obtained by subjecting the displacement percentages measured with the rhodamine sensor to PCA. The responses acquired with the fluorescein sensor did not confer significant contribution to the final grouping effect and thus were not included in PCA. The first two principal components (PC) of the analysis summarized more than 70% of the total variance in the dataset, and successfully grouped the compounds based on their structural differences, with error ellipses shown, obtained at the 95% confidence interval. For instance, the three “strongest binders” (the R–NMe_3_^+^ guests **6**, **7**, and **9**) locate in the upper-right panel, well separated from the R–NHMe_2_^+^ guests, as well as the more weakly bound R–NMe_3_^+^ guests (**8**, and **10–13**) by PC1 and PC2. Most of the R–NHMe_2_^+^ guests (**14–17**) locate close to each other, indicating that our sensors are not able to discriminate them due to their weak affinity to the hosts.

**Fig. 8:**
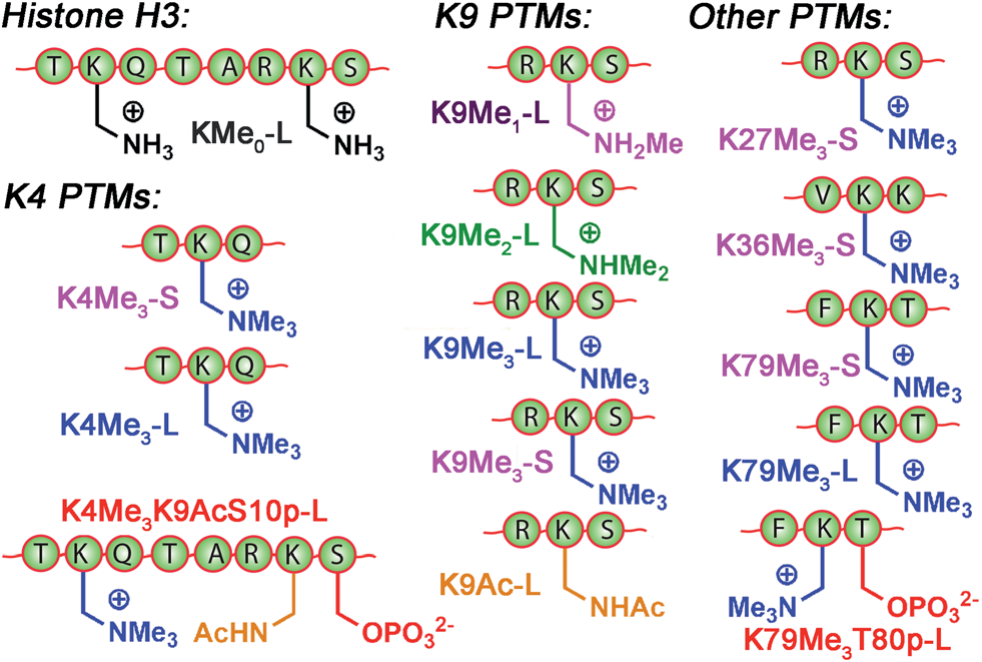
Variably modified peptides used in this study. L = 20/21 amino acid residues. S = 10–15 amino acid residues. See ESI† for full peptide sequences.

**Fig. 9:**
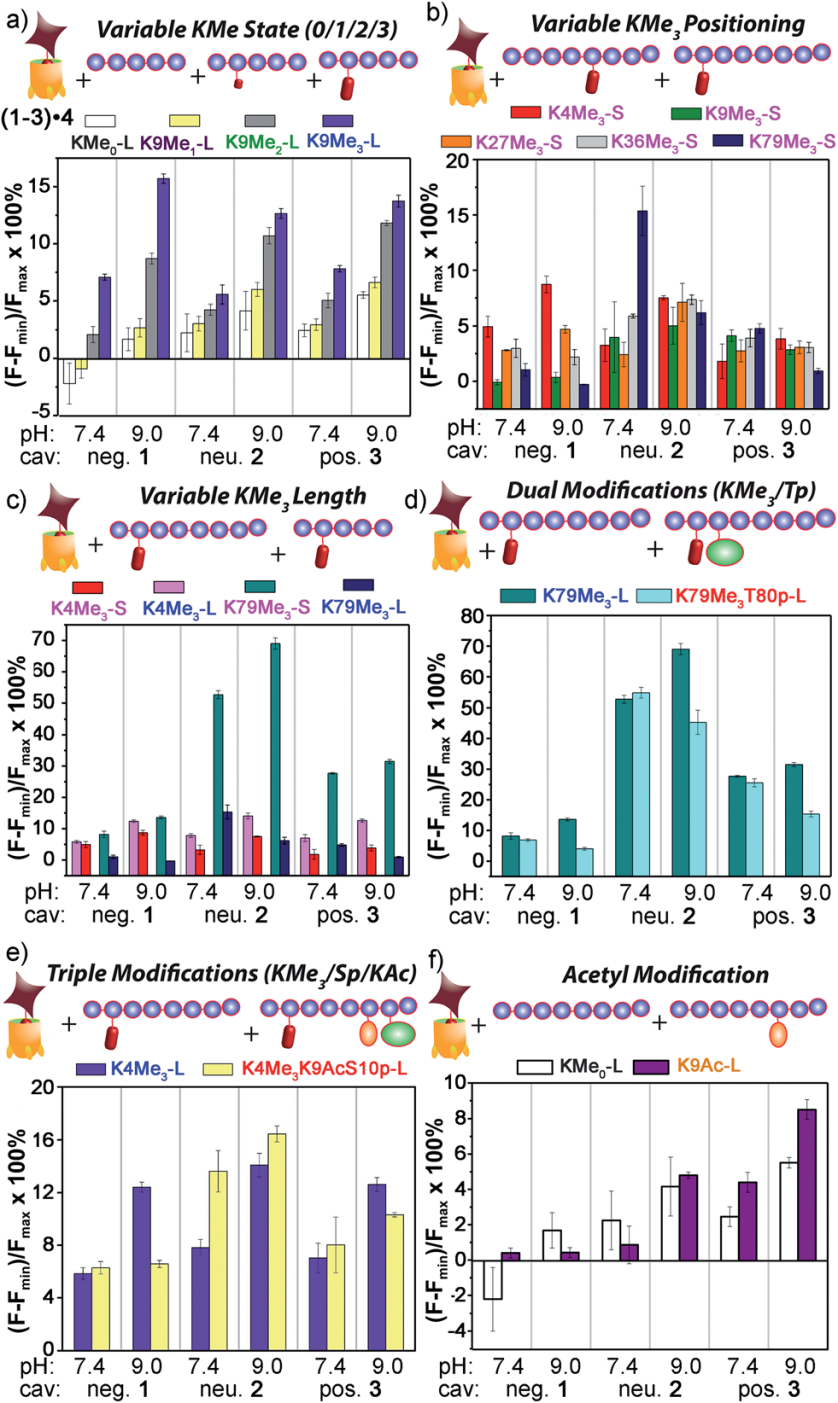
Peptide discrimination *via* fluorescence recovery. Percent displacement plots grouped the peptides based on (a) Lys methylation state; (b) Lys methylation site; (c) length; and presence of other modifications, such as (d) nearby phosphorylation; (e) remote acetylation and phosphorylation; and (f) Lys acetylation in the absence of methylation. For negative **1**, [guest **4**] = 3 μM and [cavitand **1**] = 4 μM; and for neutral **2** and positive **3**, [guest **4**] = 3 μM, [cavitand **2** or **3**], = 5 μM. [peptide] = 10 μM.

**Fig. 10:**
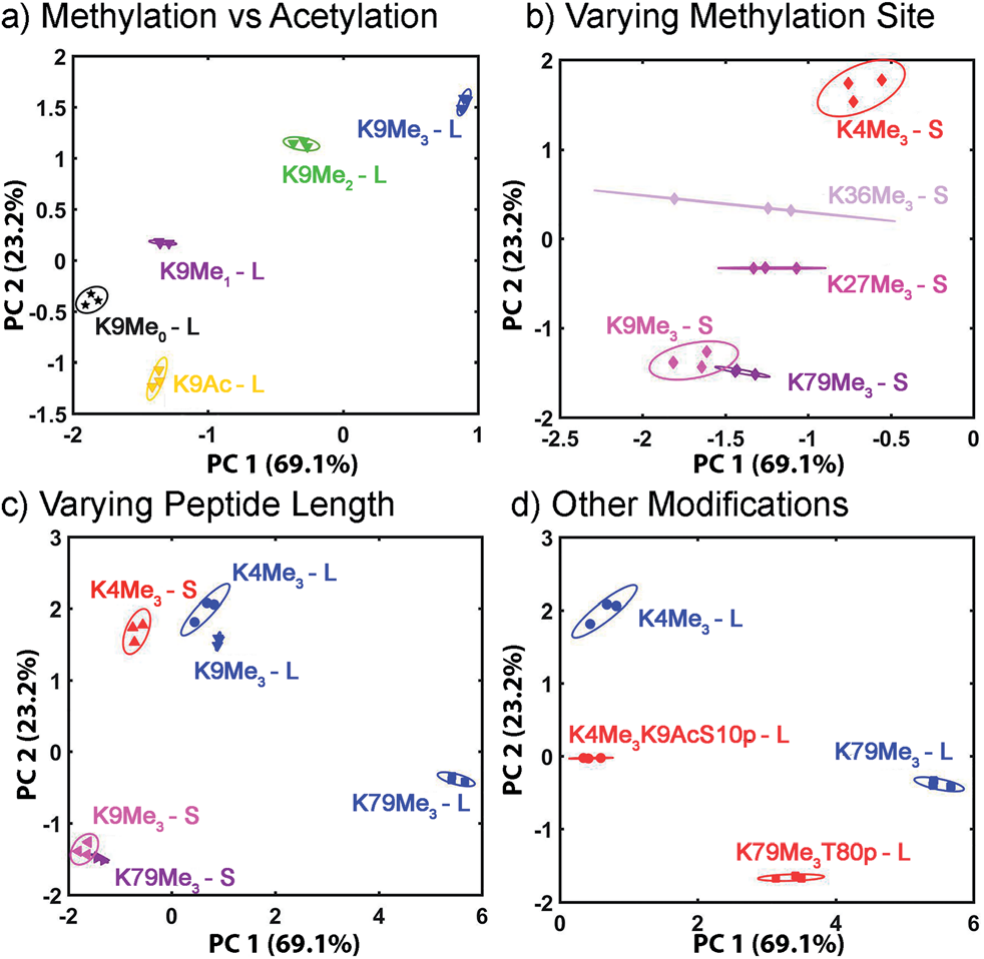
Peptide discrimination PCA. Zoom-in scores plot for peptides (a) with various degrees of methylation or acetylation; (b) with varying methylation sites; (c) with different peptide lengths; (d) with phosphorylation and/or acetylation near the trimethylated site. The error ellipses were obtained at 95% confidence interval.

The small molecule screen illustrates the potency of the sensor system in detecting small differences in guest structure. By incorporating 3 different cavitands in 4 different pH environments, discrimination can be achieved between groups as similar as OH/NH_2_, or even two different R_2_NH_2_^+^ groups that display identical cavity binding handles. The sensitivity to non-cavity-based effects such as aggregation, H-bonding and charge matching is an encouraging sign of the array's ability to sense remote differences in target structure, not merely cavity-based recognition of a specific group. We next applied this sensor array to discriminate between various PTMs from peptides derived from histone H3. The 14 peptides (see Table S-3† for full peptide sequences) are illustrated in Fig. 8, and provide a variety of challenges for discrimination by the sensor array. Some of the variations are quite large, such as varying the methylation state at K9 from 0/1/2/3 methyl groups (K9Me_0_–K9Me_3_). Other differences are more subtle, including changing the position of the trimethylation PTM on the backbone. Five major methylation sites K4, K9, K27, K36, and K79 were analysed. The overall size of the peptide chain could be varied while retaining identical methylation level and position. For example, both 21 (denoted as long, L) and 11 (short, S) amino acid fragments of K4Me_3_, K9Me_3_ and K79Me_3_ were tested. Finally, non-methylation PTMs were tested including lysine acylations and serine/threonine phosphorylations, including the presence of remote dual or triple modifications on the same peptide. As the small molecule study showed weak response at acidic pHs, the peptide assay was performed in neutral (pH 7.4) and basic (pH 9.0) conditions only. In addition, as RhB guest was the most effective, the peptide assay only involved cavitands **1–3** and guest **4**. Side-by-side comparisons of the fluorescence recovery for the various different peptide groupings is shown in Fig. 9, and the corresponding PCA analysis in Fig. 10.

The initial test was the simplest: discriminate between varying methylation levels at the same position (Fig. 9a). As predicted by the small molecule screen, the sensors showed excellent selectivity for different lysine methylation levels. The affinity of the tested peptides was far stronger than the small molecules, and only 100 μM peptide was added to the **1–3·4** sensors (as opposed to 1 mM small molecule) to give the responses shown in Fig. 9. The control H3K9 peptides (1–21) displaying zero and mono-methylation at the K9 position caused negligible displacement with all of the cavitand **1–3**·RhB guest **4** sensors at this concentration. In contrast, as much as 10% and 15% of guest **4** was displaced by the di- (K9Me_2_) and tri-methylated (K9Me_3_) peptides at pH 9.0. The largest signal difference between K9Me_2_ and K9Me_3_ was observed with the negative cavitand **1**, although all three hosts showed significant discrimination.

The more challenging task is to discriminate identical modifications in different positions, and this is shown in Fig. 9b, using the short peptides (10–15 amino acids) with trimethylation PTMs at K4, K9, K27, K36, and K79. Cavitand **3** was not particularly effective at discriminating between these positionally variable KMe_3_ modifications, but the other hosts were far more successful. The **1·4** sensor effectively discriminated three trimethylation sites at both pH 7.4 and 9.0, with the percent displacement decreasing between K4 > K27 > K36. However, negligible response was observed for K9Me_3_-S and K79Me_3_-S. Fortunately, the largest percent displacement in this case (∼15%) was obtained with K79Me_3_ and the neutral cavitand **2** at pH 9.0. While individual cavitands are not capable of fully discriminating the five different positional variations by themselves, the combination of the three hosts is successful.

These signal differences are interesting, as they illustrate the effects of remote structure on the recognition process. For example, the lysine residues at K9 and K27 share the same adjacent amino acids with the sequence of –RKS–, yet show significant displacement differences, especially with **1**. In addition, whereas the long, 21 amino acid K9Me_3_ gave strong fluorescence recovery with **1·4**, the short 15 amino acid K9Me_3_ showed no displacement of **4** and much lower affinity for **1**, despite the immediately adjacent amino acids and the KMe_3_ binding target being identical. This suggests that other, remote factors are important components of the displacement, and those factors affect the differently functionalized hosts differently.

The remote variations in peptide property suggested that the assay could be able to discriminate between identical modifications, at identical positions, in peptide fragments of varying length, as shown in Fig. 9c. Short and long versions of K4Me_3_ and K79Me_3_ were tested, and again, a wide disparity in the effectiveness of the different cavitands was observed. In this case, anionic cavitand **1** showed quite poor selectivity, but cationic **3** and especially neutral **2** showed exceptional discrimination between the different length peptides. K79Me_3_-L (a.a. 69–89) caused almost 70% displacement of **4** from **2** at pH 9.0. This affinity is all the more remarkable for the fact that the short K79Me_3_-S (a.a. 73–83) peptide shows almost zero displacement of **4** from **2**, despite the only variations in structure occurring remotely, over 6 amino acid residues distant from the host interaction! The K4Me_3_ variants could be slightly discriminated, but far less effectively so.

As well as detecting remote changes in peptide structure, the sensor system can detect the presence of other, non-methylation modifications remote from the KMe_3_ site, as shown in Fig. 9d and e. Two multiply modified peptides were compared with their single KMe_3_ equivalents. K79Me_3_ contains an adjacent threonine residue, and so K79Me_3_-L was tested against K79Me_3_T80p-L. In addition, K4Me_3_-L was compared with K4Me_3_K9AcS10p-L. This variant has no differences in adjacent residues, but incorporates acetyl and phosphoryl groups at residues 9 and 10, remote from K4Me_3_. Threonine phosphorylation in K79Me_3_T80p-L decreased its affinity to all cavitands at pH 9.0 with respect to K79Me_3_-L, most probably resulting from repulsion between the cavitand and the negatively charged phosphate group. The charge of the phosphate group appears not to be the overriding control factor, as the drop in signal was greatest for cavitand **2**: presumably the larger OPO_3_^2–^ group provides a steric barrier to target binding.

These results indicate that the large, structurally variable peptides have more complex interactions with the hosts than the small molecules **6–18**. While the selectivity for KMe_3_ is consistent with cavity-based recognition (Fig. 9a), a solely host:guest type interaction does not explain the sensitivity to peptide length, and suggests other factors are in play. These variables are most likely due to either (or both) charge and hydrophobicity variations. For example, K27Me_3_-S has much higher hydrophobicity than the other short peptides used here (*i.e.* K4/9/36/79Me_3_-S), as reflected by its GRAVY score, *i.e.* the hydrophobicity index for peptides calculated from the amino acid composition (see ESI Table S-3†). Hydrophobicity is not the only global factor to be considered: the pI of the peptides varies as well. K79Me_3_-S has the lowest pI (4.56) among the 14 peptides tested and carries negative charge at neutral and basic pHs, while the other peptides have pIs close to or larger than 10 and are positively charged under the conditions of the assay. The specific examples from Fig. 9c can also be compared: K79Me_3_-L (a.a. 69–89) has a far higher hydrophobicity and positive charge (GRAVY = –0.518, pI = 9.98) than K79Me_3_-S (a.a. 73–83, GRAVY –0.927, pI 4.56), commensurate with the large differences in displacement observed, especially with the **2·4** sensor. The less well-distinguished K4Me_3_ pair are far more similar in charge/hydrophobicity as their pI and GRAVY scores are far closer (12.83, –1.448 for K4Me_3_-L, 12.02, –1.890 for K4Me_3_-S).

To provide a more accurate description of these unusual selectivities, fluorescence titration experiments similar to those performed for small molecules **6–9** were performed on the K79Me_3_ peptide pairing shown in Fig. 9d (*i.e.* K79Me_3_T80p-L and K79Me_3_) and neutral cavitand **2** (see Experimental section and Fig. S-25†). Interestingly, curve fitting analysis that assumes a 1 : 1 binding model was unsuccessful for these peptides. In contrast, the peptide curves fit very well to the Hill equation, indicating that a multivalent interaction is occurring, with multiple cavitands binding to a single peptide. The analysis shows that phosphorylation at the adjacent amino acid does not change the microscopic association constant *k* between the peptide and host, but alters the binding cooperativity. The *n* value for K79Me_3_-L binding to host **2** is 3.1, indicating positive cooperativity, but that of K79Me_3_T80p-L reduces to 0.6, indicating negative cooperativity. The much large *n* value leads to the larger apparent K for peptide-cavitand binding. A similar phenomenon was also observed for binding between the long di- and trimethylated K9 peptides, K9Me_2_-L and K9Me_3_-L, to cavitand **1**, in which the *n* value changes from 2.3 to 0.8.

The low solubility of the cavitands (especially **2**) in water precludes analysis of exactly where the second interaction is occurring. These hosts are well-known to bind a range of lipophilic small molecules,^22^ so interactions with exposed phenylalanine or leucine sidechains would be most likely. Other small molecule hosts show selectivity for Phe residues, so hydrophobic targeting is plausible, albeit much weaker than the NMe_3_^+^ residue binding. We have also previously shown that host **1** can bind proteins at membrane bilayer interfaces *via* charge-based interactions.^28^ As the neutral cavitand **2** is the host that is most capable of non-KMe_3_ recognition, however, it is most likely that hydrophobic association is the dominant factor here.

If the secondary modifications occurred on remote sites, as in K4Me_3_K9AcS10p-L, the effect on target affinity was less predictable, but even more pronounced. The combination of acetylation and phosphorylation reduces the overall charge of the peptide, and K4Me_3_K9AcS10p-L shows increased displacement of **4** from neutral cavitand **2** when compared to K79Me_3_-L. This trend is similar to what observed between the low pI peptide H3K79Me_3_-S and the other high pI peptides in Fig. 9b: the drop in peptide positive charge increased the percent displacement with the neutral cavitand. The effect was reversed with anionic cavitand **1** at pH 9.0, however, with K79Me_3_-L showing stronger affinity than K4Me_3_K9AcS10p-L. Again, varying the nature of cavitand has often contradictory effects on the affinity of remotely varied peptides. The origin of the selectivity is not always immediately apparent, but combining these effects into a single array allows for exquisite, almost antibody-like reading of epigenetic markers in histone fragments.

These results introduce another question: our original assumption was that the cavitand required a substrate with an NMe_3_^+^ (or at least NMe_2_H^+^) group to effect displacement of **4**. Can the system detect non-methylation PTMs? This was tested with the control H3 peptide and H3K9Ac (Fig. 9f). In this case, no NMe_3_^+^ groups are present at all, but the two peptides display different charge states in solution. Interestingly, cavitand **1** was incapable of discrimination, but cavitands **2** (at pH 9.0) and **3** (at both pH values) showed observable, albeit small, differences in fluorescence recovery between H3 and H3K9. These more hydrophobic hosts were far more sensitive to peptide hydrophobicity, and the multivalent association modes allowed remote discrimination between targets.

The displacement plots were also analysed *via* PCA (see Fig. 10 and ESI†). The large impact on the target binding due to peptide size causes a clustering effect in the full peptide PCA panel (see ESI† for full PCA plot). This full screen uses the signals from all cavitands **1–3**, with guest **4**, at pH 7.4 and 9.0. In this global screen, the short trimethylated peptides locate on the same panel as the long, non-, mono-, di-methylated, or acetylated peptides. The first PC summarizes more than 75% of the overall variance of the data set, PC 1, shows exceptional discrimination between that peptide series and the trimethylated long peptides. If PCA was carried out on separated groups of peptides, *i.e.*, the long and short groups, clear separation of peptides carrying different levels of methylation and varied modifications was achieved on the scores plots (Fig. 10). Separation of different methylation states was simple and clear, as expected (Fig. 10a). Methylation state is easily discriminated by both PC 1 and PC 2 (Fig. 10b). For the long peptides (Fig. 10c), the trimethylated peptides locate on the right panel, separated from those with lower levels of methylation or acetylation by PC1, which represents the major trend of the data and indicates that our array is most powerful at discriminating different methylation levels, as expected. The vertical axis, PC 2, is most effective at separating different modification positions: little variation in PC 1 is observed for K4/9/27/36/79Me_3_, but they are highly variable in PC 2. In addition, PC 2 is most effective at separating other modifications (Fig. 10d). Additional phosphorylation and/or acetylation also moves the peptide downward, *i.e.* their PC2 values become more negative. The subtle changes in the displacement data from Fig. 9 are easily distinguished in the PCA, illustrating the power of the sensing system for small changes in peptide PTMs.

## Conclusions

In conclusion, we have shown that variably functionalized self-folding deep cavitands are capable of highly selective discrimination between substrates containing small, or remote structural differences. Multiple different factors contribute to this discrimination: the hosts contain both a deep, electron-rich cavity that is capable of selective R–NMe_3_^+^ binding and charged upper rim functional groups, conferring dual-mode selectivity on target recognition. By pairing the hosts with strongly bound fluorescent indicators, a pH responsive fluorescence displacement assay can be created, which combines variable fluorophore affinity with variable guest binding in different pH conditions to provide a highly sensitive assay. The lipophilic nature of the hosts also introduces a self-aggregative quenching phenomenon that adds an additional variable to the arrayed sensor. Principal component analysis provides a simple method of target discrimination. The range of targets that can be analysed is extensive: small molecules with single atom variations in structure can be differentiated, centred around a R–NMe_3_^+^ motif.

The system is most effective when analysing histone peptide post-translational modifications. By employing an arrayed suite of different host molecules, positionally selective recognition of peptide PTMs is possible. The hosts are capable of discriminating between different lysine methylation states, as expected, but are also affected by remote changes in peptide hydrophobicity and overall charge, allowing differentiation of identical methylation PTMs at varying positions on the peptide. Varying adjacent amino acid residues can be discriminated by the combined sensor array, allowing detection of variables remote to the targeted binding site, and this effect can be extended to the detection of non-methylation PTMs such as phosphorylation or acetylation. In addition, the sensor is affected by global changes in structure, so is capable of discriminating between identical PTMs, at identical positions on amino acid fragments that vary only in peptide backbone length. Binding selectivity for small molecule synthetic receptors is usually limited to recognition of the targeted function, but in this arrayed system, the synergistic application of multiple variables allows dual-mode deep cavitands to approach levels of recognition selectivity usually only seen with natural antibodies. Further applications of this sensor system to the recognition of protein modifications in cellular extracts are underway in our laboratories.

## Experimental

### General

Molecular modelling (semi-empirical calculations) was performed using the AM1 force field using SPARTAN. Cavitands **1** (ref. 23*b*) and **2**,^24^ and guest **5** (ref. 25) were synthesized according to literature procedures. See ESI† for synthesis and characterization of new molecules (**3**, **4**, **9**, **10**, **13**). Solvents were dried through a commercial solvent purification system (Pure Process Technologies, Inc.). All histone H3 (purity > 95%) peptides were purchased from Anaspec and used as received. All curve fittings were performed with Origin 8.0. PCA was performed with XLSTAT (Addinsoft) with default settings. Scores plots with error ellipses were created in MatLab.

### Measurement of fluorescence quenching and guest–cavitand binding

The quenching assay was carried out by mixing 10 μL of the fluorescent guest **4** (30 μM), 10 μL of the cavitand (**1**, **2**, or **3**) (0–400 μM), 70 μL of the incubation buffer at a selected pH in the 96-well plate, adding water to bring the total volume up to 100 μL, and incubating with mild shaking for 15 min. Variable pH was obtained by adding 70 mM (final concentration in the mixture) sodium salt of citrate (pH 3.3), phosphate (pH 5.0 and 7.4), and carbonate (pH 9.0). The fluorescence signal (*F*) was recorded in a Perkin Elmer Wallac 1420 Victor 2 Microplate Reader (PerkinElmer) with the Ex/Em wavelengths at 530/605 nm. Dissociation constants were obtained by the Stern–Volmer equation,^29^ with *F*_0_ being the fluorescence with no cavitand:*F*_0_/*F* = 1 + 1/*K*_d_ [cavitand]

### Small molecule and peptide screening and *K*_i_ calculation

The fluorescence displacement assay was conducted with a 96-well plate. Each well contained the sensor solution – a mixture of 10 μL of the fluorescent guest **4** (30 μM), 10 μL of the cavitand (**1** at 40 μM; **2** or **3** at 50 μM), and 70 μL of the buffer (pH 3.3, 5.0, 7.4, and 9.0 obtained with the same buffer components as described above). Then 10 μL of H_2_O (as the control for obtaining the minimum fluorescence, *F*_min_), the peptide (100 μM), or the small molecule guest (1 mM) was added to each well. There were also wells containing guest **4** or **5** under the exact buffer environment but no cavitand or competitor added, for measurement of *F*_max_. The fluorescence was acquired after 15 minutes' incubation under mild shaking. For small molecules, the displacement assay was also performed with the sensor formed between the cavitand **1** and fluorescent guest **5** at pH 7.4 and 9.0, following the exact same procedure.

To achieve the fluorescence recovery curves for *K*_d_ measurement of selected competitors, *i.e.* the small molecule **6–9**, or the peptide series of H3K9 (K9Me_0_, Me_1_, Me_2_, and Me_3_) and H3K79 (K79Me_3_ and K79Me_3_T80p), 10 μL of the competitor solution was added to the sensor solution (at pH 7.4 or 9.0) to obtain the final concentration of 0–5 mM (for small molecules) or 0–20 μM.

Calculation of the *K*_d_ of the small molecule guest **6–8** followed the typical approach for determination of inhibitor binding constant in protein-ligand-inhibitor binding assays:^30^ firstly, the titration curve of fluorescence against small molecule concentration were obtained; then the IC_50_ value (the “inhibitor” concentration giving half maximum response) was obtained by fitting the curve to the exponential decay equation:*F* = *A* exp(–[guest]/*t*_1_)where IC_50_ = ln(1/2)*t*_1_. At last, the *K*_i_ value which is equivalent to the dissociation constant of the complex formed by the small molecule and the cavitand was obtained by the following equation:*K*_i_ = IC_50_/([L]_50_/*K*_d_ + [cavitand]_0_/*K*_d_ + 1)where [L]_50_ is the concentration of the free small molecule at 50% inhibition (approximated to be the starting small molecule concentration), [cavitand]_0_ is the cavitand concentration at 0% inhibition, and *K*_d_ is the dissociation constant for the cavitand **1**–guest **4** complex.

To evaluate peptide binding to the cavitand, we assumed one peptide could bind to multiple cavitands, and the equilibrium constant *k* was obtained by fitting with the fluorescence *vs.* [peptide] to the Hill equation:*F* = *F*_min_ + (*F*_max_ – *F*_min_) × [peptide]^*n*^/(*k* ^*n*^ + [peptide]^*n*^).

The “n” represents binding cooperativity.
