# Supplementary material for: Site selective reading of epigenetic markers by a dual-mode synthetic receptor array
Source: Chem Sci. 2017 Mar 22;8(5):3960–70. doi: 10.1039/c7sc00865a (PMC5433514; doi:10.1039/c7sc00865a)
Supplement: Supplementary file 1 [file SC-008-C7SC00865A-s001.pdf]

Electronic Supplementary Material (ESI) for Chemical Science.
This journal is © The Royal Society of Chemistry 2017

Site Selective Reading of Epigenetic Markers by a Dual-Mode Synthetic Receptor Array

Yang Liu,[2] Lizeth Perez,[1] Magi Mettry,[1] Adam D. Gill,[3] Samantha R. Byers[1], Connor J. Easley,[1] Christopher J. Bardeen,[1] Wenwan Zhong[1,2] and Richard J. Hooley[1,3]**

[1]Department of Chemistry; [2]Environmental Toxicology Program; [3]Department of Biochemistry and Molecular Biology; University of California-Riverside, Riverside, CA 92521, U.S.A.

richard.hooley@ucr.edu; wenwan.zhong@ucr.edu

Electronic Supplementary Information

Table of Contents:

1. General Information *S*-2
2. Experimental Procedures *S*-2
    - Synthesis of New Molecules *S*-2
    - NMR spectra *S*-7
    - Mass spectra data *S*-11
    - Other Experimental Procedures *S*-14
3. Supporting Figures *S*-15
    - Rhodamine B Guest 4 Optical Properties *S*-15
    - Small Molecule Displacement Data *S*-17
    - Peptide Displacement Data *S*-21
4. References *S*-23

1. General Information

^{1}H and ^{13}C NMR spectra were recorded on either a Varian Inova 400 MHz NMR spectrometer, or a Bruker 500 MHz NMR spectrometer. All NMR spectra processed using MestReNova by Mestrelab Research S.L. Proton (^{1}H) chemical shifts are reported in parts per million (δ) with respect to tetramethylsilane (TMS, δ=0), and referenced internally with respect to the protio solvent impurity. ^{13}C chemical shifts are reported in parts per million (δ) and referenced internally with respect to ^{12}C solvent signal. Deuterated NMR solvents were obtained from Cambridge Isotope Laboratories, Inc., Andover, MA, and used without further purification. Mass spectra were recorded by electrospray ionization on an LTQ-XL linear ion trap mass spectrometer (Thermo Scientific, San Jose, CA). MALDI spectra were obtained using an AB Sciex TOF/TOF 5800 MALDI mass spectrometer with positive ion mode. Infrared spectra were recorded on a Bruker Alpha FTIR spectrometer. Melting points were collected using the melting point apparatus from Stanford Research Systems (SRS) Digimelt MPA 160. All materials were purchased from Sigma Aldrich (St. Louis, MO), Fisher Scientific (Fairlawn, NJ), or TCI (Tokyo, Japan) and were used as received.

2. Experimental Procedures

Synthesis of New Molecules:

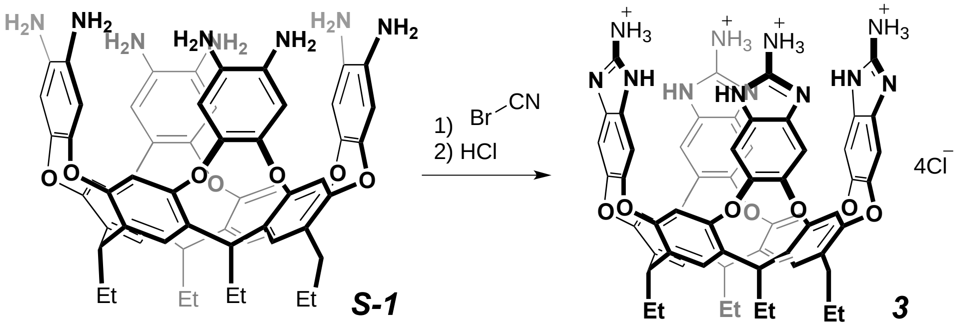

Ammonium Cavitand 3: Following a procedure slightly modified from published methods:[1] Octaamine cavitand ***S-1*** (454 mg, 0.44 mmol) in EtOH was stirred for 15 min at room temperature under N_2 atmosphere. A solution of cyanogen bromide (300 mg, 2.83 mmol) in EtOH was added drop wise over 15 min. The reaction mixture was stirred for 24 h at room temperature. The mixture was then cooled to 0 °C and basified to pH ~9. Solvent was removed by rotatory evaporation. The solid was redissolved in MeOH and 2 drop of 1M HCl was added, a white precipitate was formed.

The cavitand was obtained as red solid (232 mg, 45% yield). ^{1}H NMR (500 MHz, DMSO-d_6), δ: 12.1 (s, 8H), 8.7 (s, 8H), 7.79 (s, 4H), 7.74 (s, 8H), 7.65 (s, 4H), 5.33 (t, *J* = 7.8 Hz, 4H), 2.24 (m, 8 H), 0.85 (t, *J* = 7.2 Hz, 12H); ^{13}C NMR (125 MHz, DMSO-d_6) δ: 155.9, 152.0, 148.7, 135.6, 127.1, 125.3, 117.1, 107.4, 35.7, 25.2, 19.8, 12.4; m.p. > 300 °C (decomp). MALDI-MS: $C_{64}H_{53}N_{12}O_8^+$ [M - 4Cl - 3H]$^+$: expected: 1117.4104; found: 1117.3110. Data is consistent with previous publication.[1]

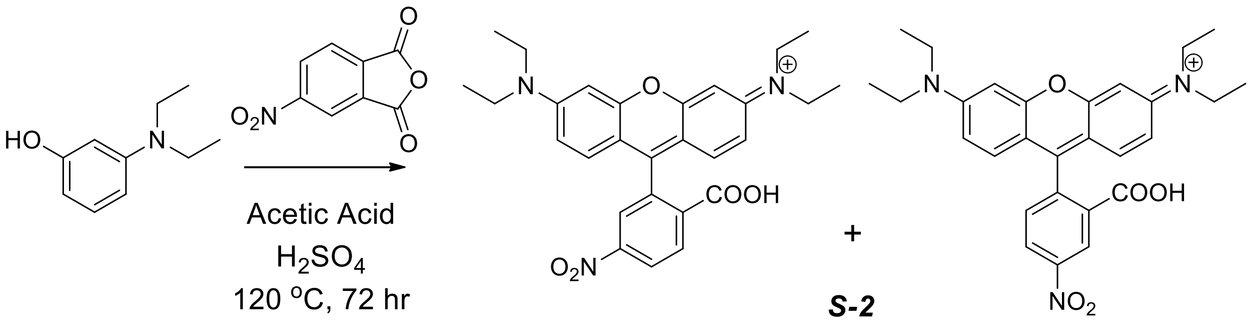

5/6 Nitro Rhodamine B S-2: To a stirred solution of 3-diethylaminophenol (1.0 g, 5.18 mmol) in glacial acetic acid (100 ml) was added concentrated H_2SO_4 catalyst (5-drops) followed by 4-nitrophthalic anhydride (1.71 g, 10.36 mmol). The reaction mixture was refluxed for 72 h. After cooling, the reaction was diluted into water (300 ml) and extracted with DCM (30 ml × 3). The solvent (DCM) was removed *in vacuo* to afford a mixture (47:53 %) of 5/6-nitro rhodamine B ***S-2*** (43%, 1.09g, 2.23 mmol) as a dark purple solid. ^{1}H NMR (400 MHz, $CDCl_3$, 25 °C, $CDCl_3$ = 7.26 ppm): 5-Nitro-rhodamine B: δ (ppm) = 12.33 (s, 1H), 9.11 (dd, *J* = 10.8, 2.3 Hz, 1H), 8.48 – 8.45 (m, 1H), 8.43 (dd, *J* = 8.3, 2.2 Hz, 1H), 8.24 (d, *J* = 8.6 Hz, 1H), 7.11 (d, *J* = 9.5 Hz, 1H), 6.91 (dd, *J* = 8.2, 2.1 Hz, 1H), 6.82 (t, *J* = 5.8 Hz, 1H), 6.16 – 6.10 (m, 2H), 3.35 (t, *J* = 7.1 Hz, 8H), 1.16 (q, *J* = 6.9 Hz, 12H). 6-Nitro-rhodamine B: δ (ppm) = 12.28 (s, 1H), 8.93 (d, *J* = 2.3 Hz, 1H), 8.33 (dd, *J* = 8.6, 2.2 Hz, 1H), 8.20 (t, *J* = 2.4 Hz, 1H), 7.53 (d, *J* = 8.3 Hz, 1H), 7.05 (d, *J* = 9.4 Hz, 1H), 6.75 (d, *J* = 9.1 Hz, 1H), 6.72 (d, *J* = 2.1 Hz, 1H), 6.05 (td, *J* = 9.0, 2.5 Hz, 2H), 3.37 (t, *J* = 7.1 Hz, 8H), 1.16 (q, *J* = 6.9 Hz, 12H). Data is consistent with previous publication.[1]

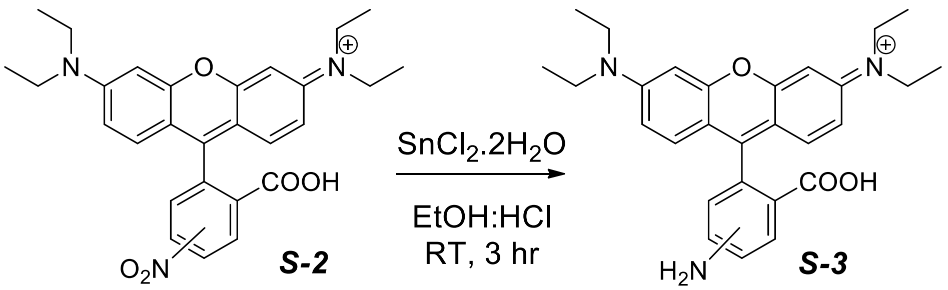

5/6 Amino Rhodamine B S-3: 5/6-nitro-rhodamine B ***S-2*** (1.09g, 2.23 mmol) was dissolved in EtOH:HCl (4:1, 50ml) along with $SnCl_2 \cdot 2H_2O$ (1.01g, 4.47 mmol). The resulting mixture was stirred at room temperature for 3 h, followed by dilution into H_2O (100 ml) and extraction with DCM (10 ml × 3). The solvent (DCM) was removed *in vacuo* to afford a mixture (51:49 %) of 5/6-amino-rhodamine B chloride as a deep magenta solid (90%, 0.92 g, 2.01 mmol). ^{1}H NMR (400 MHz, $CDCl_3$, 25 $^{\circ}$C, $CDCl_3$ = 7.26 ppm): 5-Amino-rhodamine B: δ (ppm) = 12.49 (s, 1H), 8.93 (d, *J* = 1.8 Hz, 1H), 8.46 (d, *J* = 1.9 Hz, 1H), 7.91 (d, *J* = 8.6 Hz, 1H), 6.93 (d, *J* = 9.1 Hz, 1H), 6.76 (s, 1H), 6.69 (d, *J* = 2.2 Hz, 1H), 6.14 (dd, *J* = 8.0, 2.2 Hz, 3H), 3.42 – 3.34 (m, 8H), 1.19 (dd, *J* = 11.7, 7.0 Hz, 12H). 6-Amino rhodamine B: δ (ppm) = 12.24 (s, 1H), 8.44 (d, *J* = 1.9 Hz, 1H), 7.55 (d, *J* = 8.3 Hz, 1H), 6.83 (d, *J* = 8.8 Hz, 1H), 6.74 (s, 1H), 6.67 (d, *J* = 2.3 Hz, 1H), 6.50 (d, *J* = 2.2 Hz, 1H), 6.10 – 6.02 (m, 3H), 3.42 – 3.34 (m, 8H), 1.19 (dd, *J* = 11.7, 7.0 Hz, 12H). ESI-MS: *m/z* for $C_{28}H_{32}N_3O_3$ (M^+) calculated 458.2438, found 458.2668. Data is consistent with previous publication.[1]

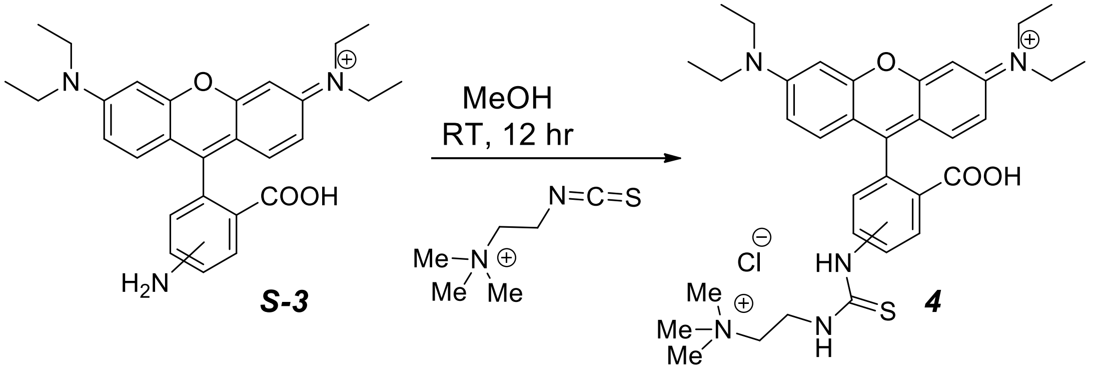

5/6 Rhodamine B guest 4: 5/6-Amino-Rhodamine B ***S-3*** (25 mg, 0.05 mmol) along with 2-isothiocyanato-N,N,N-trimethylethanaminium iodide[3] (15 mg, 0.05 mmol) was dissolved in MeOH (3 ml), and the resulting mixture was stirred at room temperature for 12 h. The solvent was removed *in vacuo* to yield the Rhodamine B Guest **4** as a bright purple solid. ^{1}H NMR (400 MHz, DMSO-d_6, 25 $^{\circ}$C, DMSO-d_6 = 2.50 ppm): Para-rhodamine B Guest (**4**): δ (ppm) = 12.59 (s, 1H),

12.23 (s, 1H), 8.65 (d, J = 2.3 Hz, 1H), 8.39 (dd, J = 6.1, 3.9 Hz, 1H), 7.70 (d, J = 8.5 Hz, 1H), 7.11 (d, J = 10.7 Hz, 1H), 6.90 – 6.85 (m, 1H), 6.81 (d, J = 3.2 Hz, 1H), 6.36 (d, J = 2.3 Hz, 1H), 6.21 (d, J = 2.4 Hz, 1H), 6.20 – 6.14 (m, 2H), 3.90 (s, 9H), 3.40 (t, J = 6.9 Hz, 2H), 3.17 (q, J = 6.9 Hz, 8H), 1.21 (t, J = 6.9 Hz, 2H), 1.10 (t, J = 6.9 Hz, 12H). Meta-rhodamine B Guest (**4**): δ (ppm) = 12.50 (s, 1H), 12.18 (s, 1H), 8.49 (dd, J = 8.3, 2.4 Hz, 1H), 7.93 (d, J = 8.5 Hz, 1H), 7.60 (d, J = 2.0 Hz, 1H), 6.97 (d, J = 9.5 Hz, 1H), 6.83 (d, J = 3.3 Hz, 1H), 6.63 (dd, J = 8.6, 2.3 Hz, 1H), 6.09 (d, J = 2.5 Hz, 2H), 6.07 (d, J = 2.4 Hz, 1H), 6.03 (d, J = 2.4 Hz, 1H), 3.90 (s, 9H), 3.38 (t, J = 6.9 Hz, 2H), 3.17 (q, J = 6.9 Hz, 8H), 1.21 (t, J = 6.9 Hz, 2H), 1.10 (t, J = 6.9 Hz, 12H). MALDI-TOF MS: *m/z* $C_{34}H_{47}ClN_5O_4S$ ($[M+Cl+H_2O]^+$) calculated 656.3032, found: 656.0713.

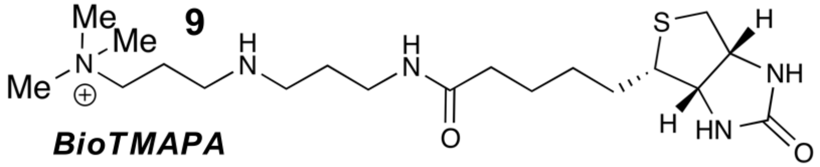

N^1-(3-biotinamidopropyl)-N^3,N^3,N^3-trimethylpropane-1,3-diaminium Iodide (BioTMAPA) 9: To a stirred solution of NHS Biotin (100 mg, 0.293 mmol) in dry THF (3 mL) under nitrogen was added N,N-dimethyl-propylenediamine (0.296 mmol). The reaction mixture was stirred at room temperature for 18 h, then concentrated via solvent removal *in vacuo*, followed by trituration with diethyl ether and hexanes. The resulting precipitate was vacuum filtered, washed with diethyl ether then hexanes, before being dried under vacuum. The solid was redissolved in dry THF (2 mL) under nitrogen, followed by the addition of iodomethane (41.6 mg, 18.2 µL, 0.293 mmol). The reaction mixture was stirred at room temperature for 4 h, then triturated with dry dichloromethane and hexanes. The precipitate was vacuum filtered, yielding a white solid (85 mg, 55% yield). ^1H NMR (400 MHz, D_2O): δ 4.63 (td, J = 8, 4 Hz, 1H), 4.46 (td, J = 8, 4 Hz, 1H), 3.47 (m, 2H), 3.35 (m, 2H), 3.32 (t, J = 7 Hz, 2H), 3.20 (s, 10H), 3.12 (t, J = 7, 2H), 3.06 (m, 4H), 2.31 (t, J = 7 Hz, 2H), 1.89 (m, 2H), 1.66 (m, 4H), 1.44 (m, 2H). ^{13}C NMR (100 MHz, DMSO-d_6): δ 172.9, 162.7, 63.7, 61.0, 59.2, 55.4, 54.9, 52.3, 45.9, 45.2, 36.2, 35.2, 28.4, 28.2, 27.8, 25.2, 21.6. ESI-MS: *m/z* $C_{19}H_{38}N_5O_2S$ for (M^+) calculated 400.6043, found 400.2982. $[\alpha]_D^{20}$ +0.073° (c 0.05, MeOH). IR 3267.65, 1649.02, 1643.39, 1455.91, 1230.49, 1073.36 cm^{-1}. Melting Point: 125 °C.

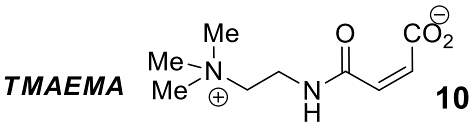

(Z)-4-oxo-4-((2-(trimethylammonio)ethyl)amino)but-2-enoate (TMAEMA) 10: Maleic anhydride (500 mg, 5.10 mmol) was added to a 100 mL round bottomed flask followed by diethyl ether (50 mL). *N,N*-Dimethylethylenediamine (5.10 mmol, 450 mg, 511µL) was then added dropwise while stirring at room temperature. After 10 min a precipitate was observed, and the resulting solid was vacuum filtered. In a 250 ml round bottom flask, 100 mg (0.540 mmol) of solid was dissolved in DMF (3 mL), followed by the addition of methyl iodide (76.6 mg, 33.6 µL, 0.54 mmol). The mixture was stirred at room temperature for 4 hours. An observed precipitate was then vacuum filtered, followed by purification via washing with diethyl ether then hexanes, to yield a yellow-white solid (125 mg, 10 % yield). ^{1}H NMR (400 MHz, D_2O): δ 6.51 (d, *J* = 12 Hz, 1H), 6.36 (t, *J* = 11 Hz, 1H), 3.80 (t, *J* = 7 Hz, 2H), 3.57 (t, *J* = 7 Hz, 2H), 3.22 (s, 9H). ^{13}C NMR (100 MHz, D_2O): δ 221.3, 220.1, 132.5, 129.8, 64.0, 53.5, 33.7. ESI-MS *m/z* $C_9H_{17}N_2O_3$ expected: 201.2429, found: $[MH]^+$ = 201.1350. IR 3427.16, 1716.51, 1628.48, 1523.53, 1476.55, 1209.11, 1077.11 cm^{-1}. Melting Point: 154 ^{o}C.

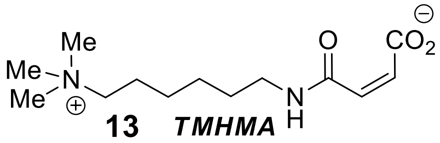

(Z)-4-oxo-4-((2-(trimethylammonio)hexyl)amino)but-2-enoate (TMHMA) 13:

To a stirred solution of maleic anhydride (170 mg, 1.73 mmol) in diethyl ether (10 mL) was added 6-(dimethylamino)hexylamine (300 µL, 1.73 mmol) dropwise. The reaction mixture was stirred at room temperature for 10 minutes, until a noticeable precipitate had formed. The resulting solid was then vacuum filtered, washed with diethyl ether, and dried. The purified solid (84 mg, 0.347 mmol) was dissolved in DMF (1 mL) along with methyl iodide (49 mg, 22.0 µL, 0.35 mmol), and the reaction mixture was stirred at room temperature for 4 h, until a precipitate had formed. The solid was then filtered, followed by purification via washing with diethyl ether then hexanes, to afford a thick orange oil (90 mg, 68 % yield). ^{1}H NMR (400 MHz, D_2O): δ 6.48 (d, *J* = 12.4 Hz, 1H), 6.30 (d, *J* = 12.2 Hz, 1H), 3.32 (m, 4H), 3.12 (s, 9H), 1.82 (q, *J* = 7 Hz, 2H), 1.59 (q, *J* = 7, 2 Hz), 1.42 (m, 2H). ^{13}C NMR (100 MHz, D_2O): δ 169.2, 167.3, 133.5, 130.3, 66.8, 53.1, 39.7,

27.8, 25.7, 25.2, 22.4. ESI-MS *m/z* $C_{13}H_{25}N_2O_3$ expected: 257.3492, found: $[MH]^+$ = 257.1549. IR 3433.54, 1710.3, 1629.09, 1561.95, 1476.44, 1216.68 cm^{-1}.

NMR Data:

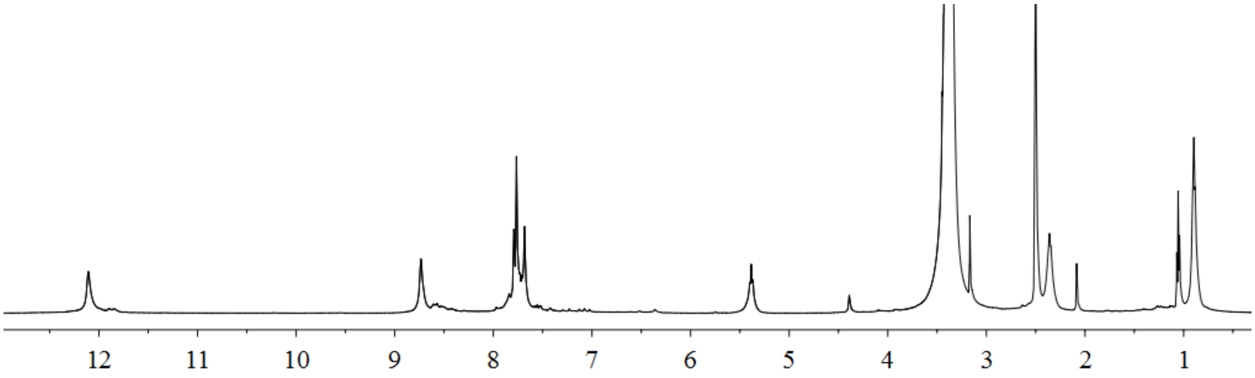

Figure S-1: ^{1}H NMR spectrum of positive cavitand **3** (500 MHz, DMSO-d_6, 64 scans, δ 14.0 – -2.0 ppm sweep width).

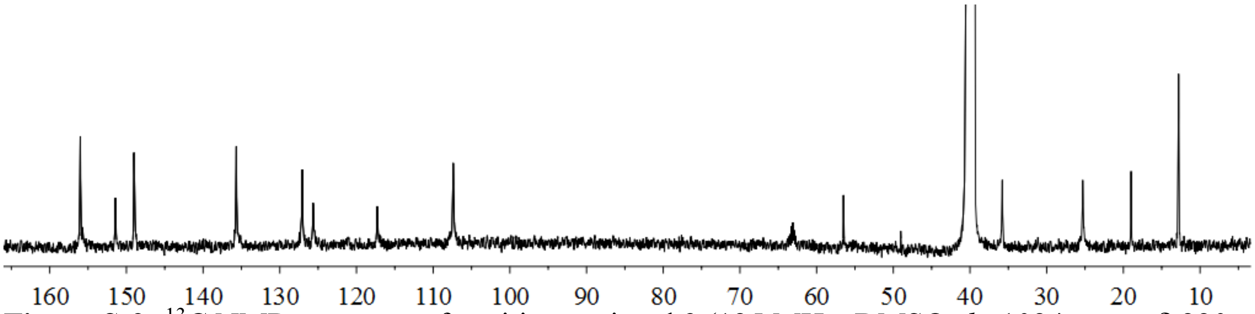

Figure S-2: ^{13}C NMR spectrum of positive cavitand **3** (125 MHz, DMSO-d_6, 1024 scans, δ 220 – -10 ppm sweep width).

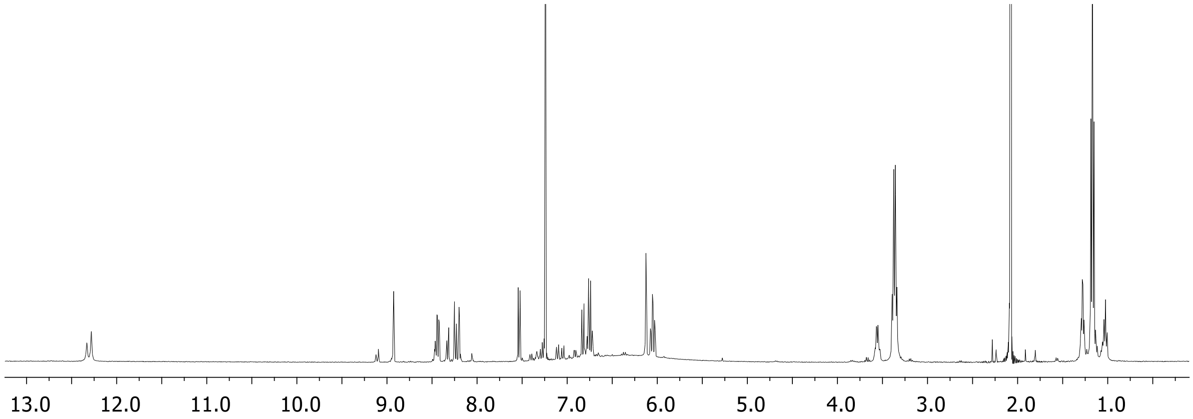

Figure S-3: ^{1}H NMR spectrum of 5/6-nitro-rhodamine B **S-2** (400 MHz, $CDCl_3$, 64 scans, δ 14.0 – -2.0 ppm sweep width).

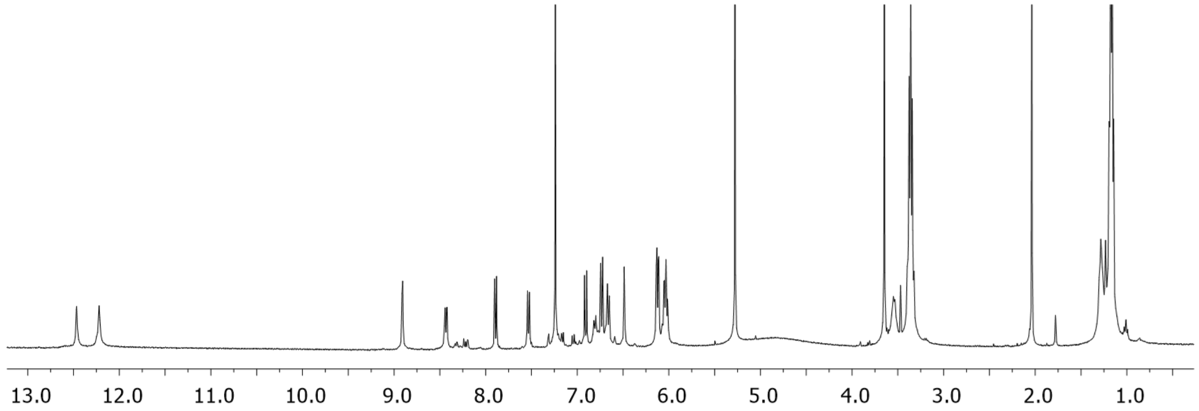

Figure S-4: ¹H NMR spectrum of 5/6-amino-rhodamine B **S-3** (400 MHz, $CDCl_3$, 64 scans, δ 14.0 – -2.0 ppm sweep width).

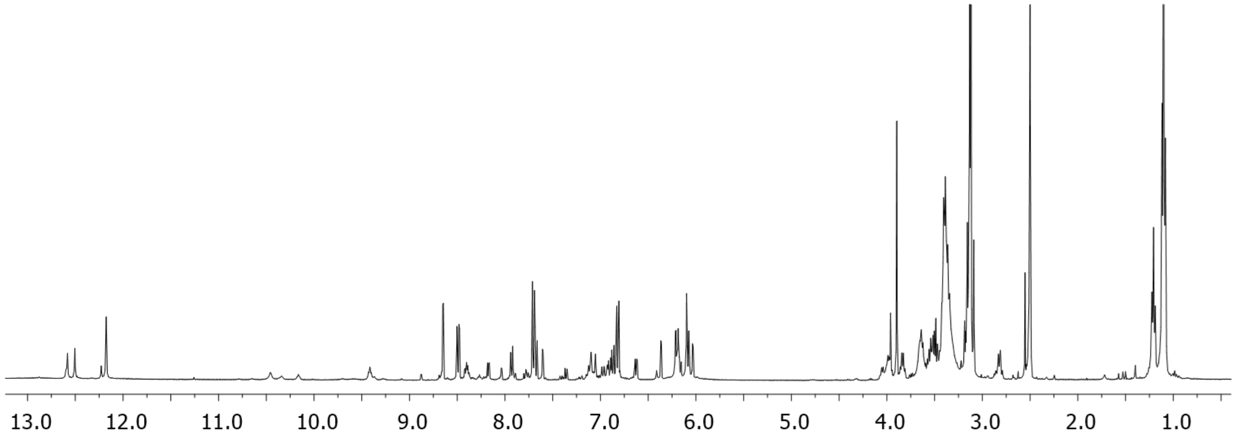

Figure S-5: ¹H NMR spectrum of rhodamine B guest **4** (400 MHz, DMSO-d_6, 64 scans, δ 14.0 –-2.0 ppm sweep width).

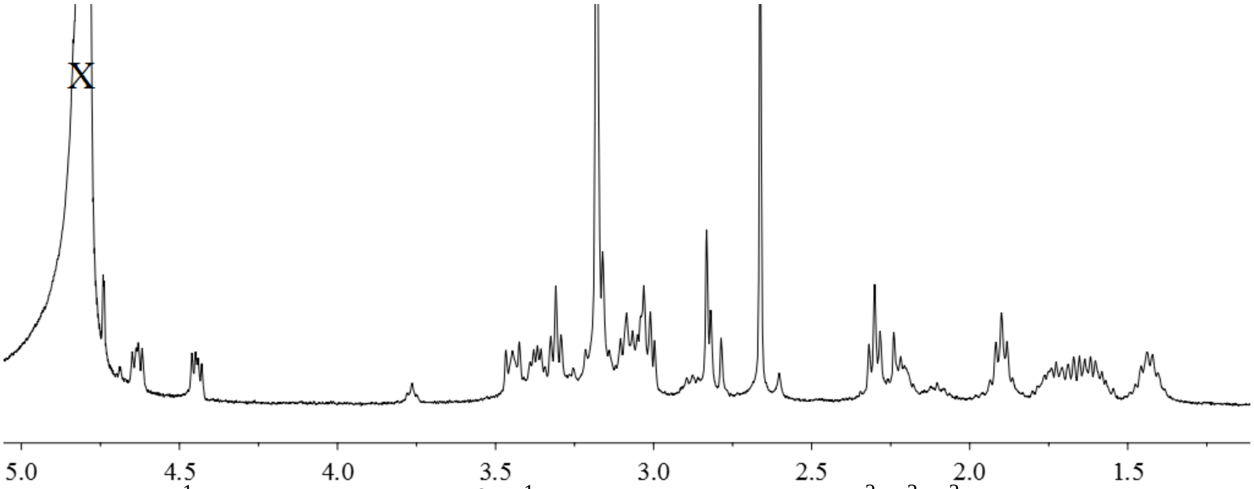

Figure S-6: ¹H NMR spectrum of N^1-(3-biotinamidopropyl)-N^3,N^3,N^3-trimethylpropane-1,3-diaminium Iodide (BioTMAPA) **9**. (400 MHz, D_2O, δ 14.0 – -2.0 ppm sweep width, 64 scans).

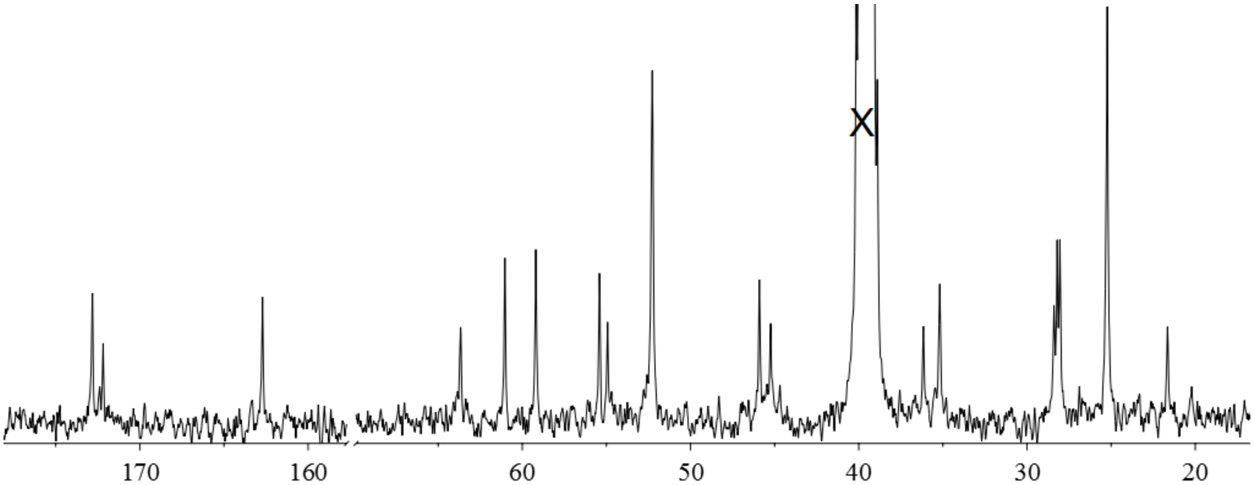

Figure S-7: ^{13}C NMR spectrum of N^1-(3-biotinamidopropyl)-N^3,N^3,N^3-trimethylpropane-1,3-diaminium Iodide (BioTMAPA) **9** (100 MHz, DMSO-d_6, δ 220 – -10 ppm sweep width, 512 scans).

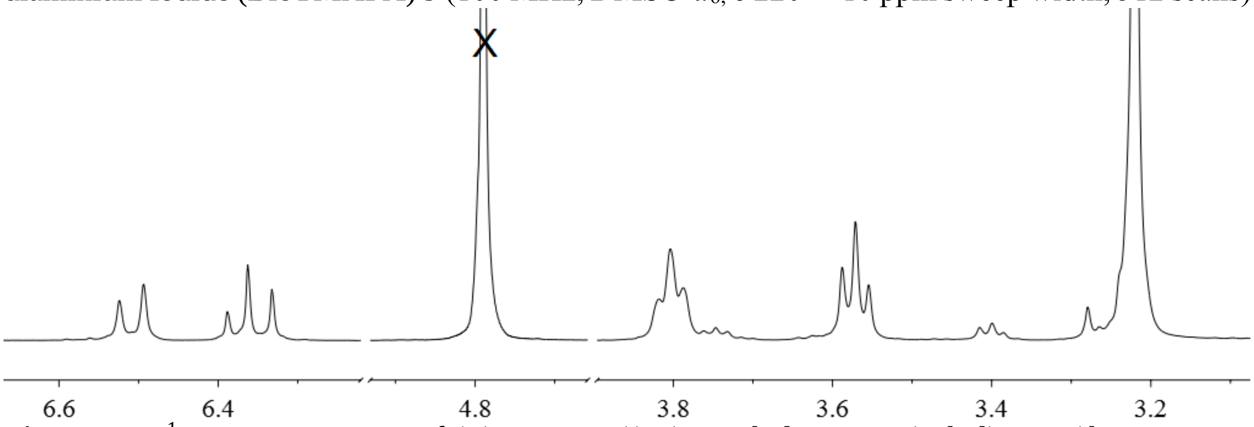

Figure S-8: ^1H NMR spectrum of (Z)-4-oxo-4-((2-(trimethylammonio)ethyl)amino)but-2-enoate (TMAEMA) **10**. (400 MHz, D_2O, δ 14.0 – -2.0 ppm sweep width, 64 scans).

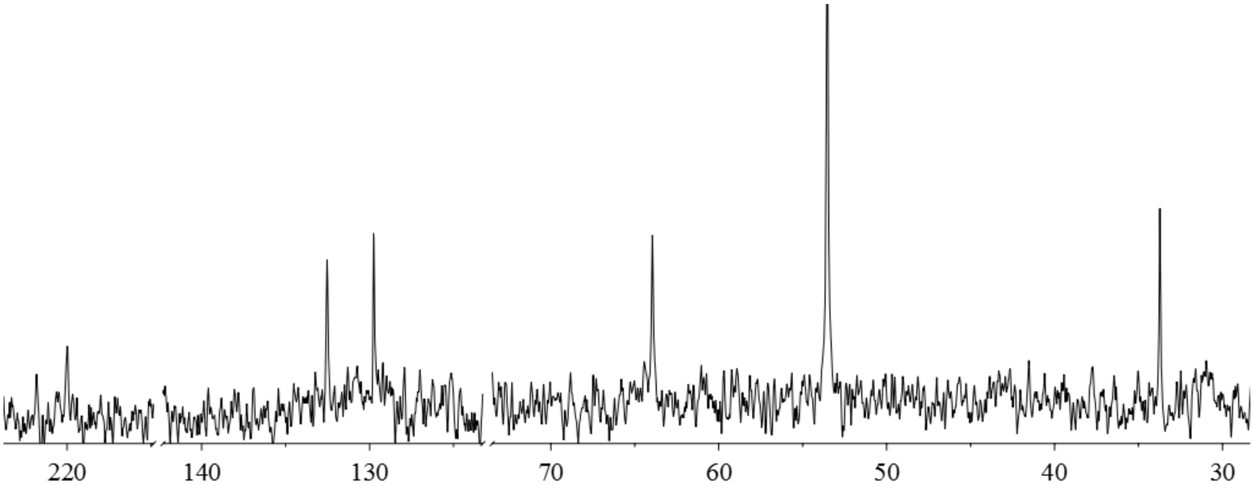

Figure S-9: ^{13}C NMR spectrum of (Z)-4-oxo-4-((2-(trimethylammonio)ethyl)amino)but-2-enoate (TMAEMA) **10** (100 MHz, $CDCl_3$, δ 220 – -10 ppm sweep width, 512 scans).

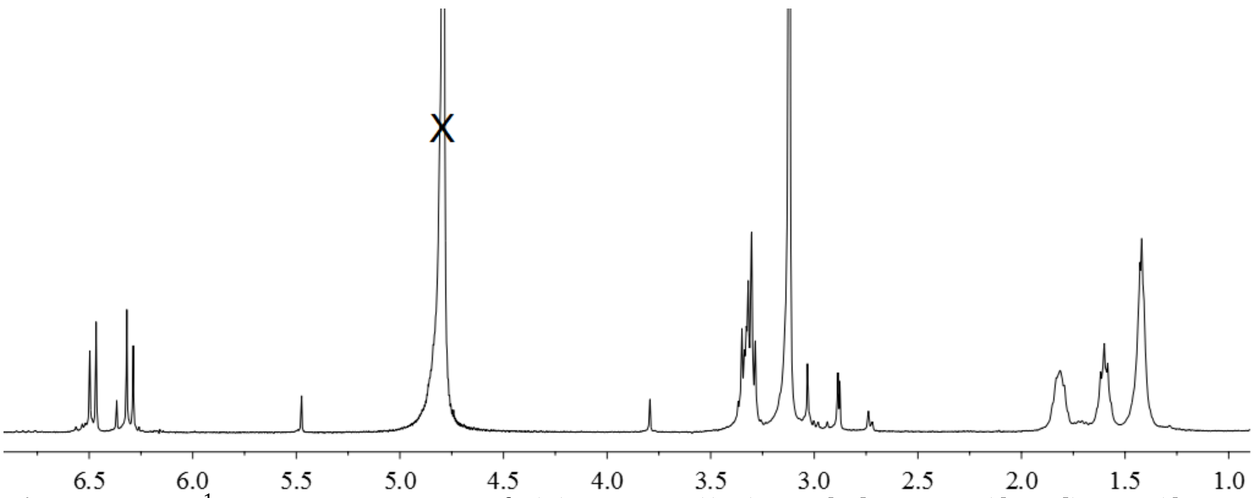

Figure S-10: ^{1}H NMR spectrum of (Z)-4-oxo-4-((2-(trimethylammonio)hexyl)amino)but-2-enoate (TMHMA) **13**. (400 MHz, D_2O, δ 14.0 – -2.0 ppm sweep width, 64 scans).

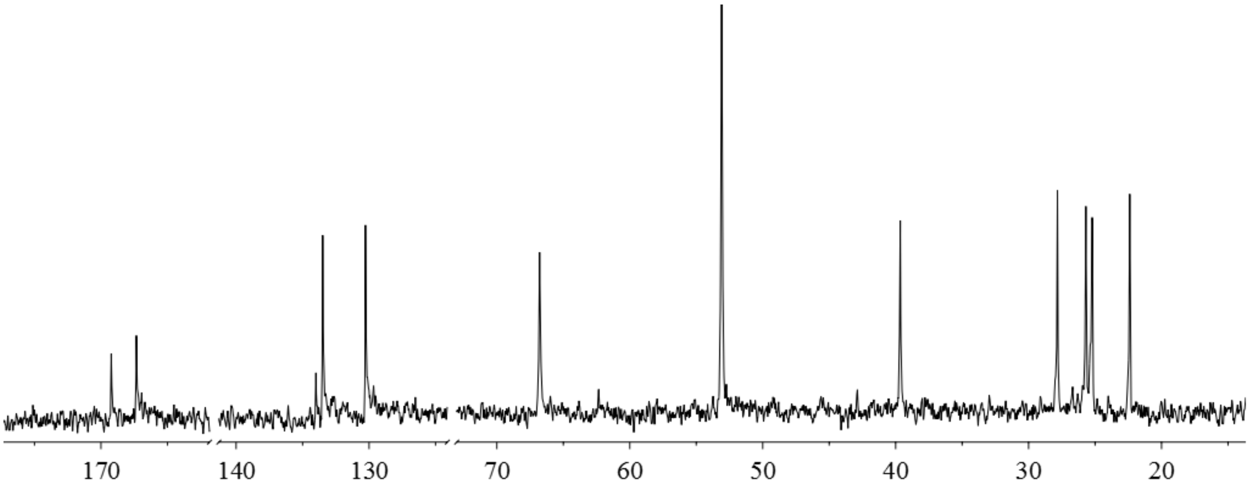

Figure S-11: ^{13}C NMR spectrum of (Z)-4-oxo-4-((2-(trimethylammonio)hexyl)amino)but-2-enoate (TMHMA) **13** (100 MHz, $CDCl_3$, δ 220 – -10 ppm sweep width, 512 scans).

Mass Spectral Data:

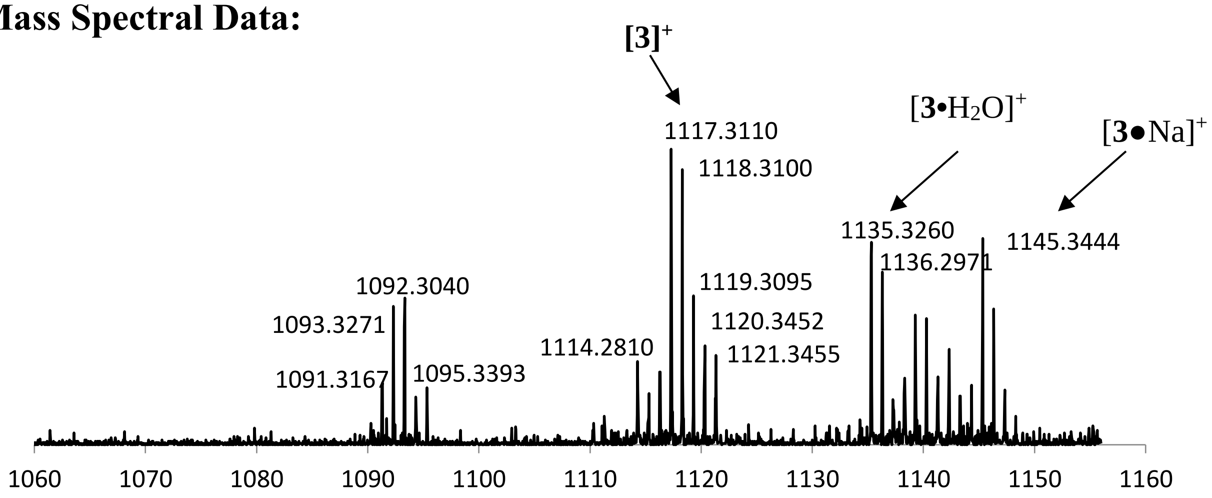

Figure S-12: MALDI-TOF MS spectrum of positively charged cavitand **3.**

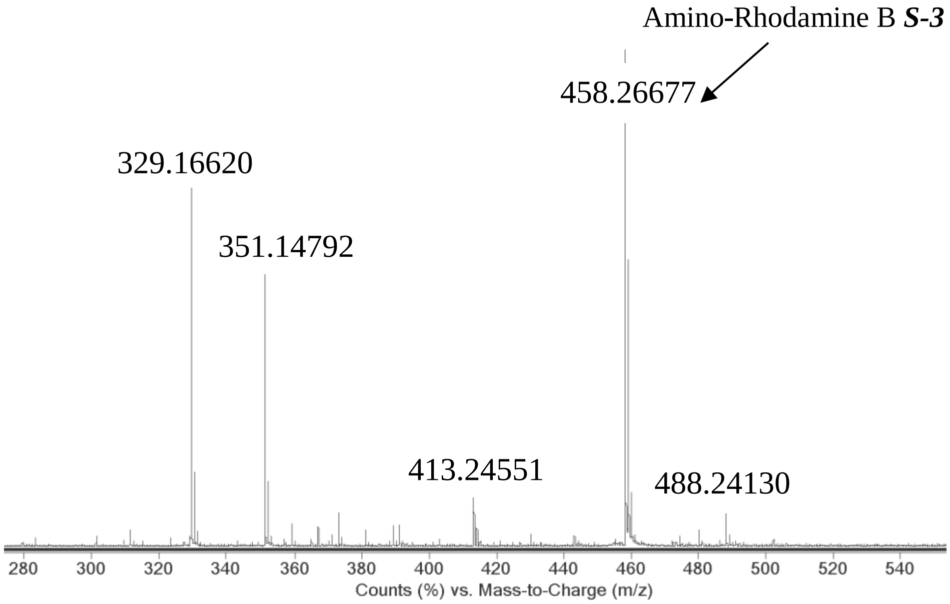

Figure S-13: ESI-MS spectrum of 5/6-amino-rhodamine B **S-3.**

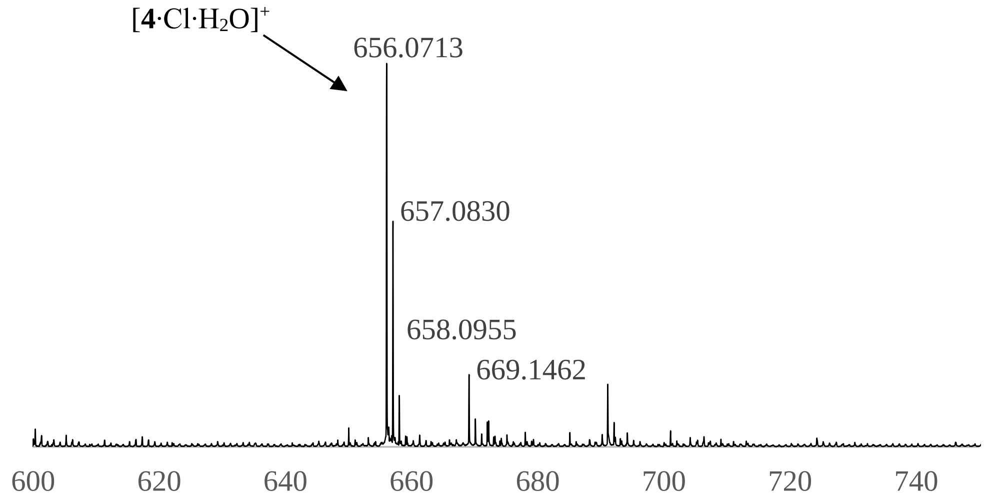

Figure S-14: MALDI-TOF MS spectrum of rhodamine B guest **4.**

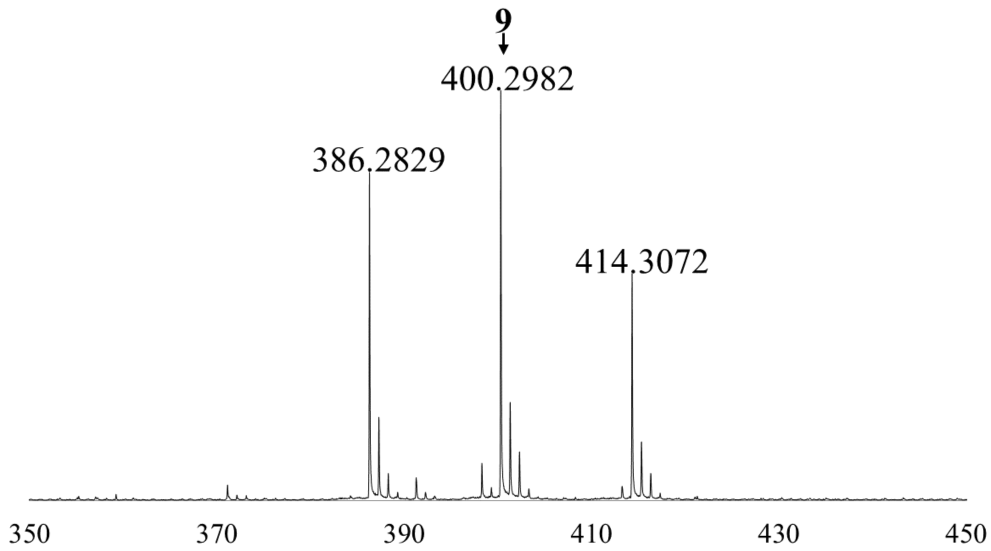

Figure S-15: ESI-MS spectrum of N^1-(3-biotinamidopropyl)-N^3,N^3,N^3-trimethylpropane-1,3-diaminium Iodide (BioTMAPA) **9**.

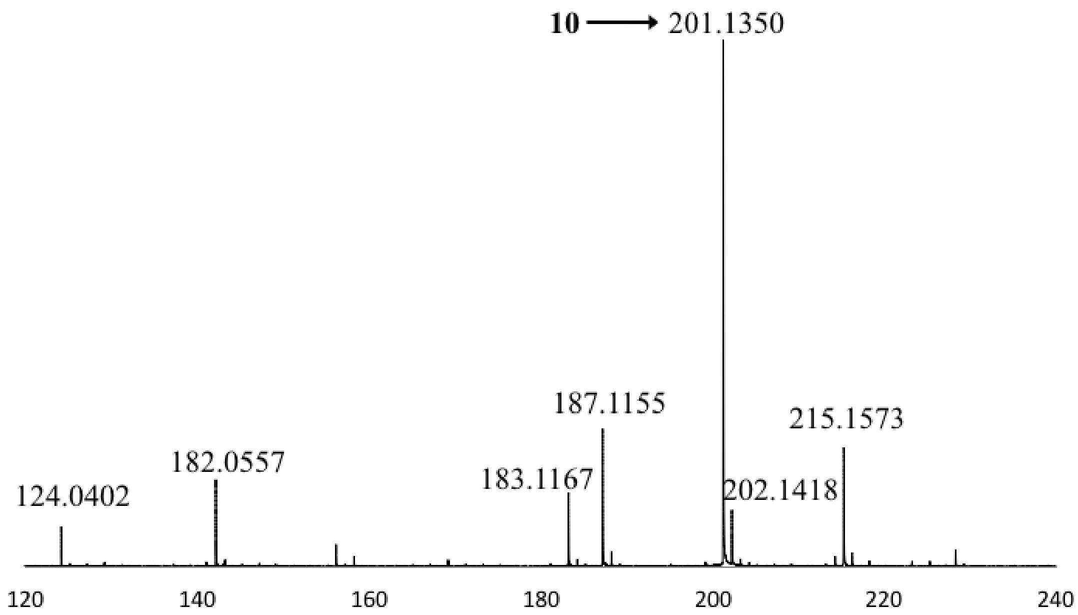

Figure S-16: ESI-MS spectrum of (Z)-4-oxo-4-((2-(trimethylammonio)ethyl)amino)but-2-enoate (TMAEMA) **10**.

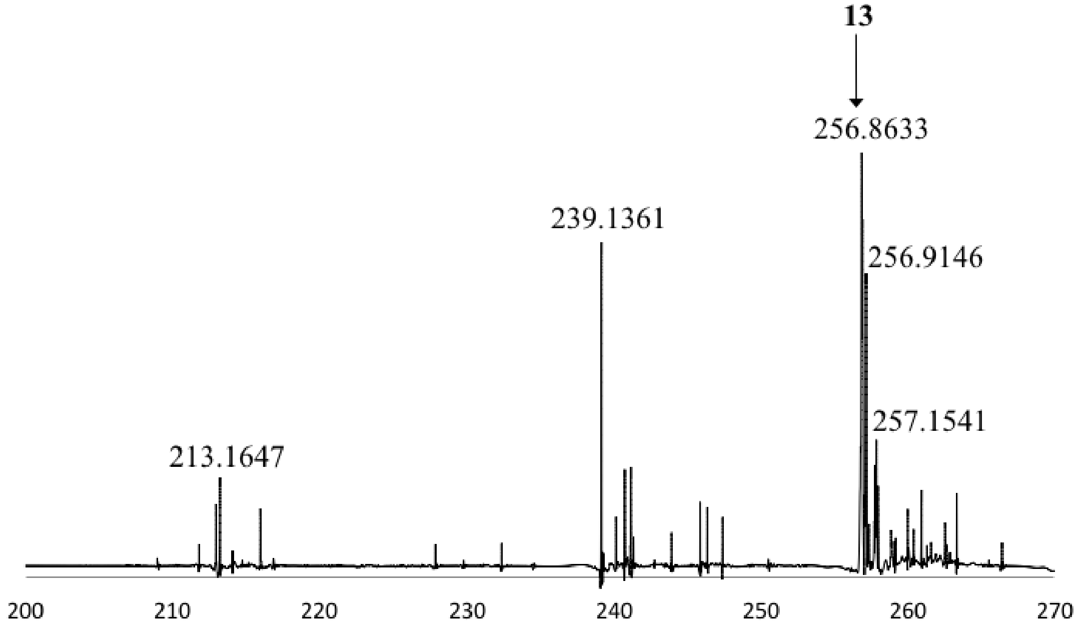

Figure S-17: ESI-MS spectrum of (Z)-4-oxo-4-((2-(trimethylammonio)hexyl)amino)but-2-enoate (TMHMA) **13.**

Other Experimental Procedures:

Determining pKa values of cavitand and small molecules

pKa values were calculated using Marvin Sketch version 16.10.10 with default s ettings. PCA was performed with XLSTAT with default settings.

Peptide displacement assay with negative cavitand:

Competition assay was carried out by first adding 10 µL Rhodamine B guest (30 µM), 10 µ L negative cavitand (40 µM), and 70 µL of phosphate buffer (pH = 7.4, 100 mM), then 10 µL of different peptides (in H_2O) were added. The fluorescent test was done after 15 minutes' incubation.

Fluorescence Life-time measurements

TrPl measurements of cavitands: Time resolved measurements were performed with a 1 kHz Coherent Libra regeneratively amplified Ti:Sapphire laser system. The 800 nm fundamental pulse was directed into a frequency doubling beta barium borate (BBO) crystal to produce the 400 nm excitation beam. The solution samples were mounted on a 1 cm cuvette stage and the emission was collected using front face detection with a 420 nm long pass filter. The emission was detected using a Hamamatsu C4334 Streakscope with 15 ps time resolution and 2.5 nm wavelength resolution. Typical laser powers were 200 - 225 microwatts.

3. Supporting Figures

Rhodamine B Guest 4 Optical Properties

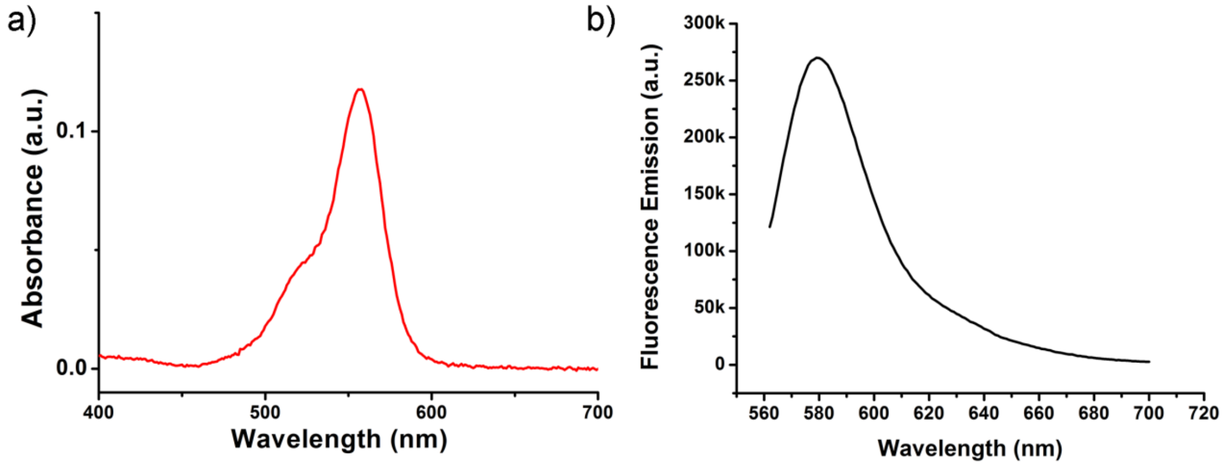

Figure S-18: a) Absorption and **b)** fluorescence emission (excitation at λ = 557 nm) spectrum of guest **4** at 3 µM in water.

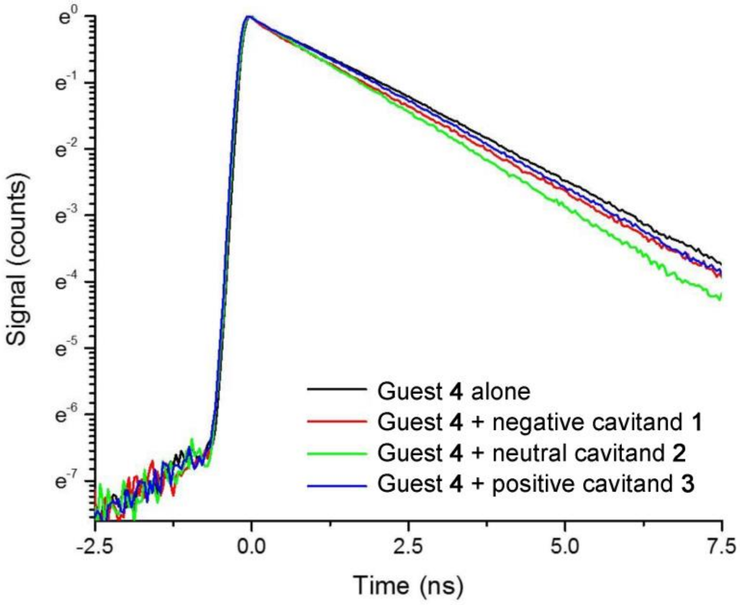

Figure S-19: Fluorescence lifetime measurement for Guest **4** at 3 µM with or without the presence of cavitand **1, 2 or 3** at 4 µM in phosphate buffer pH 7.4.

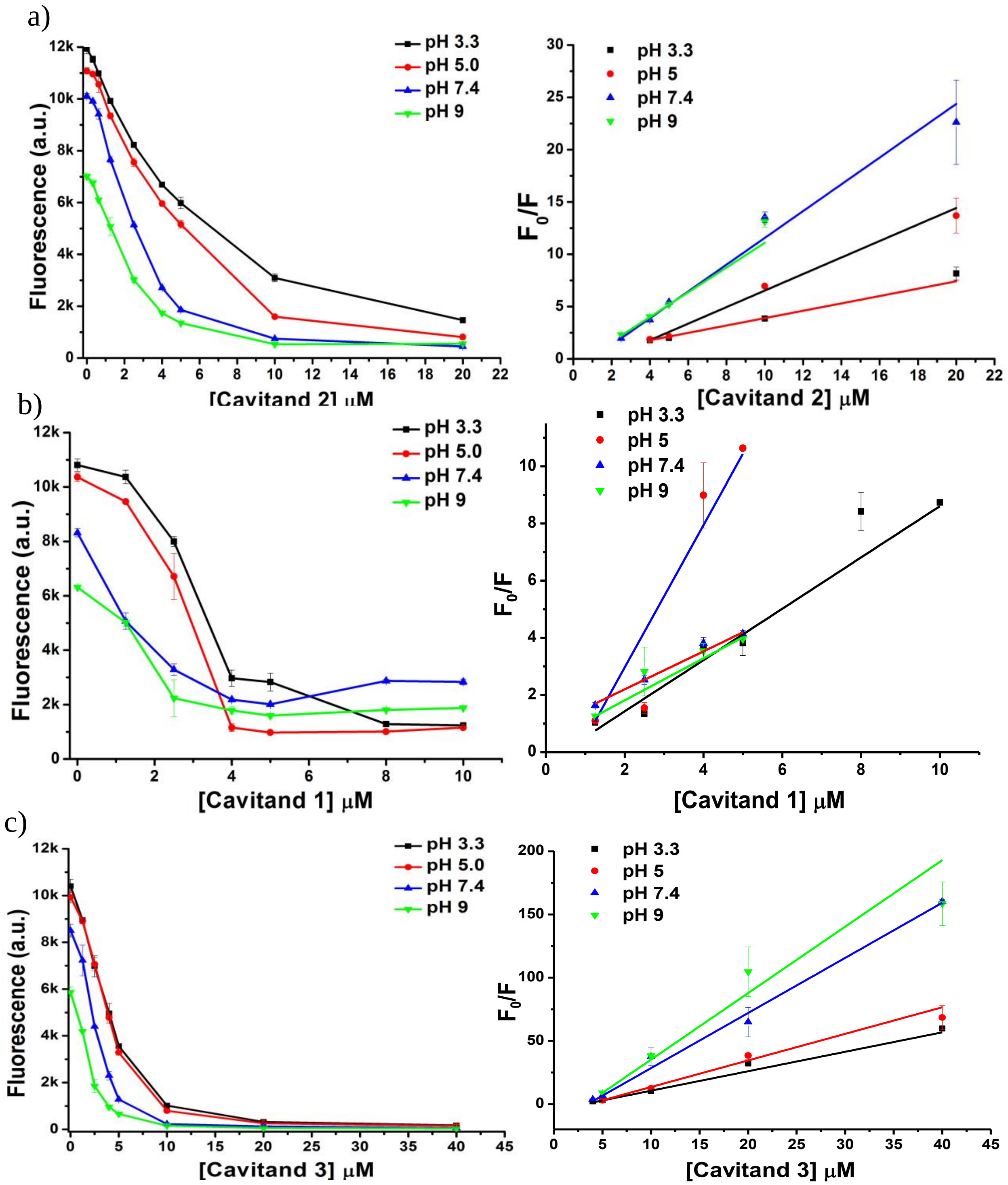

Figure S-20: The fluorescence quenching (left) and Stern-Volmer fitting (right) curves of guest **4** at 3 μM with increasing concentrations of cavitand a) **1**; b) **2** and c) **3**. The measurement was conducted in various pH: 3.3, 5.0, 7.4, and 9.0.

Small Molecule Displacement Data

Table S-1. The pKa values of the functional groups indicated by the arrows in the picture.

Guest	pKa
**8**	7.88
**9**	9.81
**10**	3.59
**12**	2.26
**13**	3.91
**14**	9.48
**17**	8.34 (terminal); 10.1 (internal)
**18**	10.35

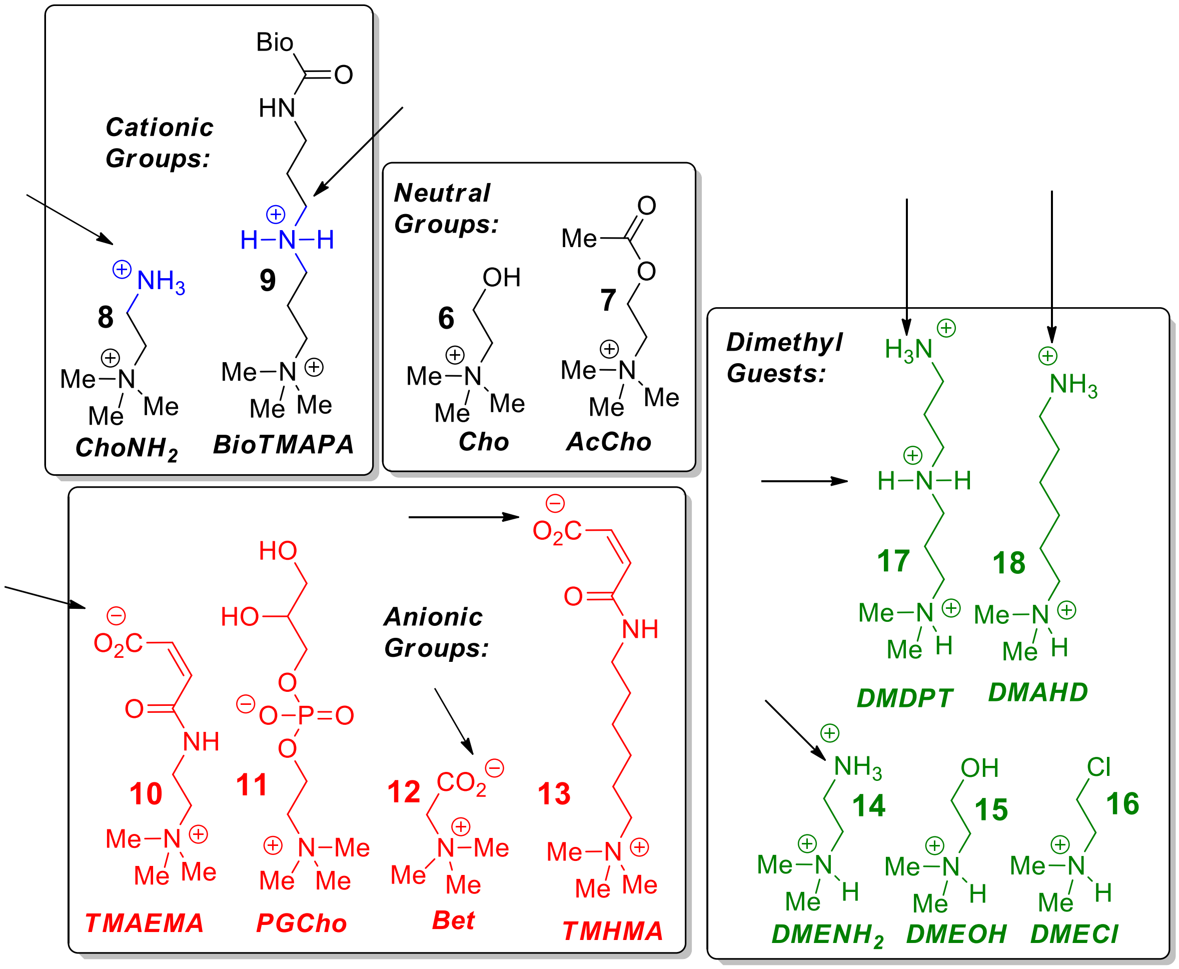

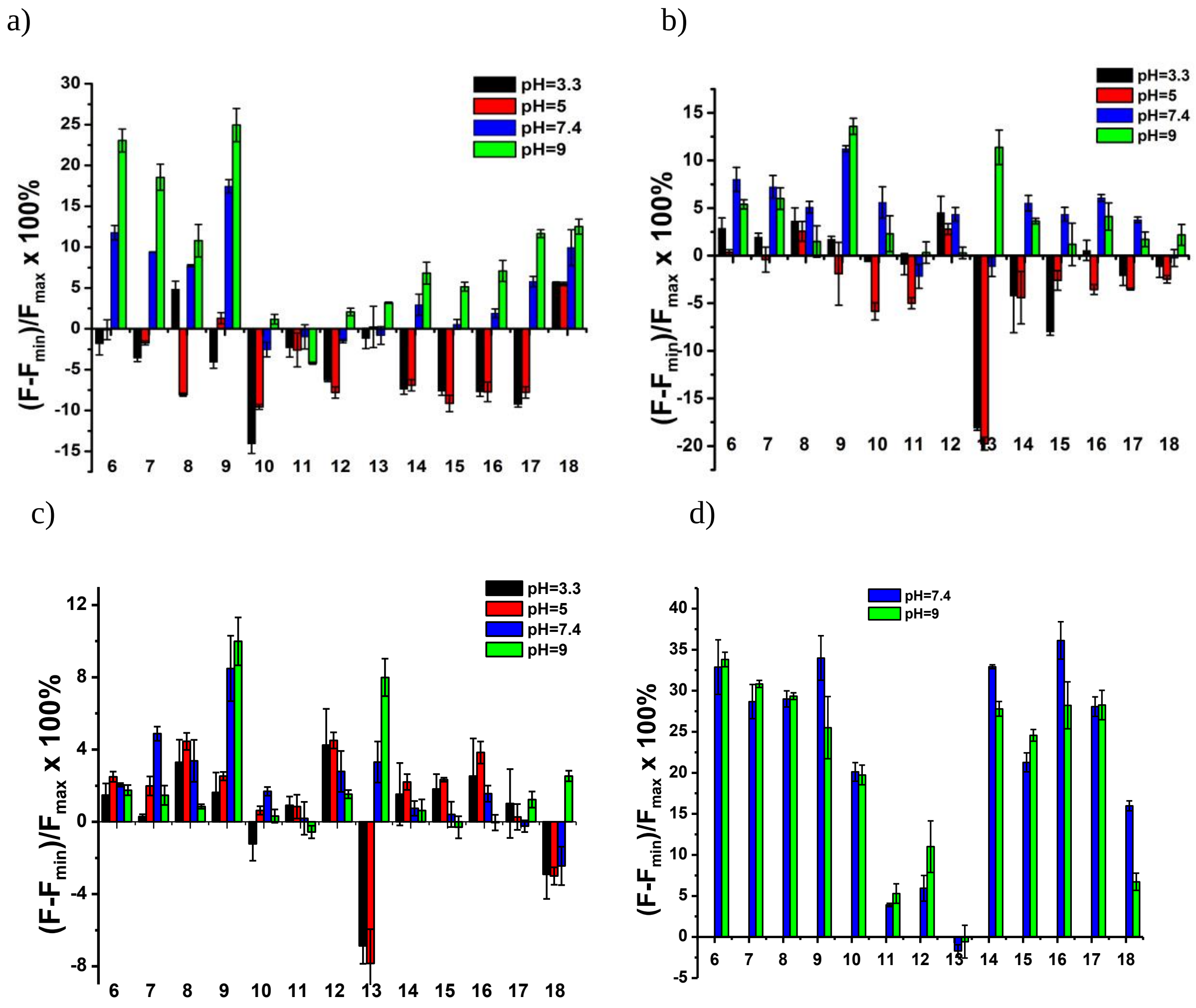

Figure S-21. The complete screening data for the small molecule guests using our fluorescent guest-cavitand array. The sensor elements were constructed by pre-incubating guest **4** at 3 µM with a) cavitand **1** at 4 µM; b) cavitand **2** at 5 µM; and c) cavitand **3** at 4 µM in the solutions of 4 pH values: 3.3, 5.0, 7.4, and 9.0. In addition, the array included the sensor formed by d) fluorescein guest **5** at 3 µM with cavitand **1** at 20 µM at pH 7.4 and 9.0.

a)

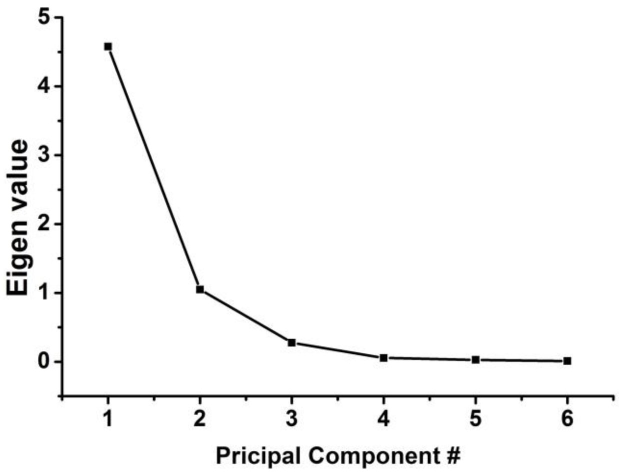

b)

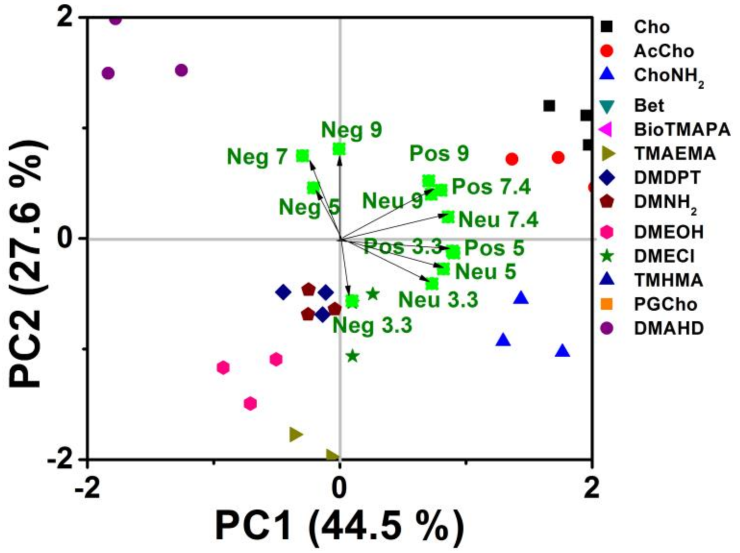

Figure S-22: a) The Scree plot of PCA on the screening data of small molecule guests, used to determine the appropriate number of principal components. After the first two components, there is a minimal change in the remaining eigenvalues, indicating that the first two components summarized the majority of the variations within the data set. b) The factor loading plot that describes the relationship between original variables and subspace dimensions. The scale was enlarged compared to the PCA plot shown in Fig. 7 to clearly show the location of each variable on this plot. From the plot, we can tell that the sensor elements at neutral and basic pH conditions had key contribution to the location of the small molecule guests at the upper panel of the score plot. Cavitands **2** and **3** contribute more to the differentiation between the R-NMe_3^+ and R-$NHMe_2^+$.

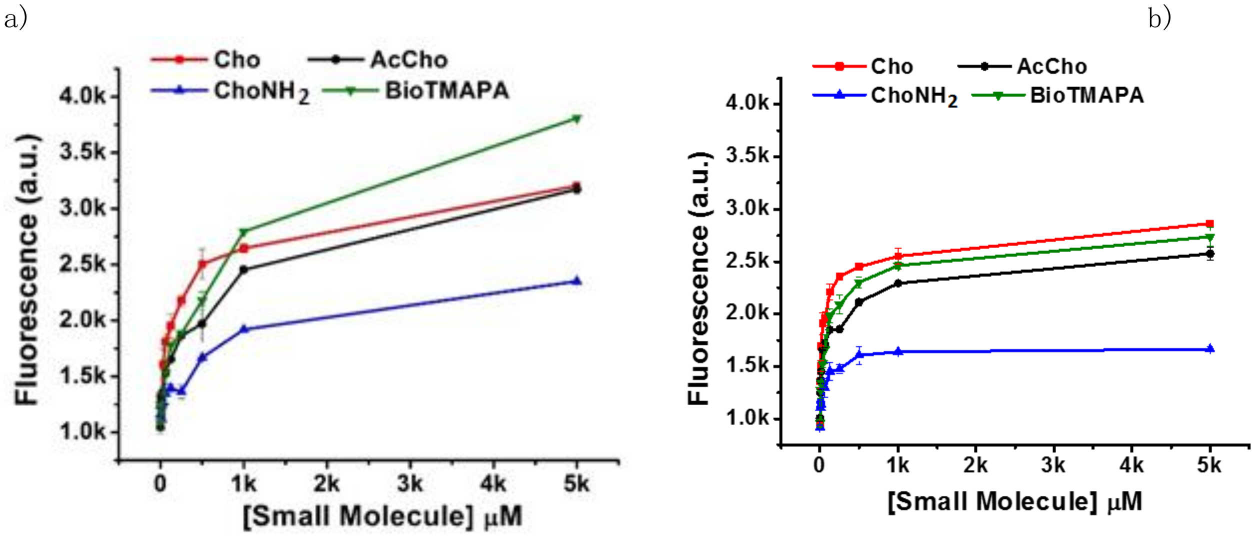

Figure S-23: Fluorescence recovery induced by mixing four selected small molecule guests at increasing concentrations from 0 to 5 mM with the sensor of **1•4** at **a)** pH 7.4 and **b)** pH 9.0.

Table S-2: The IC_{50} and K_d values of the four selected small guest molecules (**6-9**) calculated as described in Methods section from the fluorescence recovery curves shown in Fig. S6.

**IC_{50} (µM)**				
**Small molecule**	**Cho 6**	**AcCho 7**	**$ChoNH_2$ 8**	**BioTMAPA 9**
**pH 7.4**	40.9 ± 4.9	56.0 ± 8.9	66.4 ± 12.2	43.2 ± 6.6
**pH 9**	44.5 ± 6.4	49.4 ± 15.3	N/A	289.2 ± 21.9

**K_d (µM)**				
**Small molecule**	**Cho 6**	**AcCho 7**	**$ChoNH_2$ 8**	**BioTMAPA 9**
**pH 7.4**	9.9±0.6	13.9±1.6	16.5±2.4	10.5±1.0
**pH 9**	10.2±1.0	11.4±3.1	N/A	69.6±4.7

Peptide Displacement Data:

a)

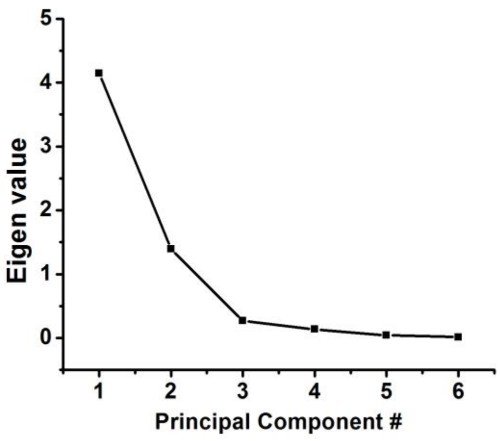

b)

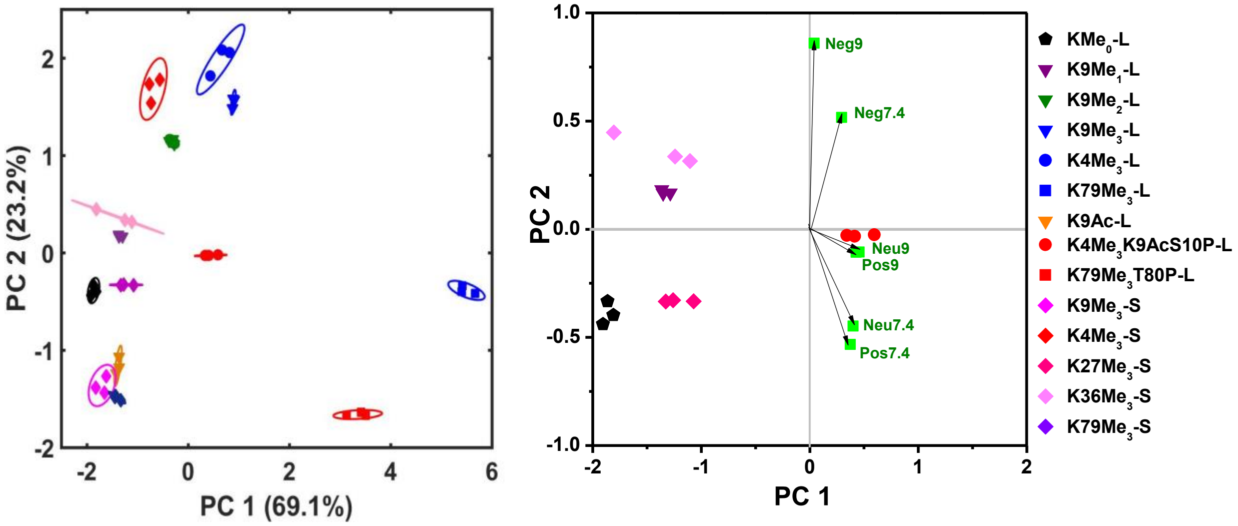

Figure S-24: a) The Scree plot and b) the score plot (left) containing all 14 peptides as shown in groups in Fig. 10, and the corresponding factor loading plot (right; with enlarged scale to clearly show the positions of all variables) of PCA for the screening data of peptides to determine of the appropriate number of principal components. We can tell from the scree plot that the first two principal components are appropriate in summarizing the major variance within the data set. The factor loading plot indicates all variables contribute to the differentiation of the trimethylated long peptides from the di-, mono-, and non-methylated long peptides, as well as from the short peptides. The sensor elements of **1•4** at pH 7.4 and 9.0, and **2•4** at pH 9.0 contribute more to the location of the peptides in the upper panel in this dimension, and the sensor elements of **2•4** at pH 7.4, and **3•4** at pH 7.4 and 9.0 contribute to the location of the guests in the lower panel. The error ellipses were obtained at 95% confidence interval.

Table S-3. The list of peptides used in our study and their sequence, pI, and GRAVY score.

No.	Peptides	Sequence (No modification shown)	pI	GRAVY
1	H3(1-21)	ARTKQTARKSTGGKAPRKQLA	12.31	-1.448
2	H3K9(Me1)(1-21)	ARTKQTARK(Me1)STGGKAPRKQLA	12.31	-1.448
3	H3K9(Me2)(1-21)	ARTKQTARK(Me2)STGGKAPRKQLA	12.31	-1.448
4	H3K9(Me3)(1-21)	ARTKQTARK(Me3)STGGKAPRKQLA	12.31	-1.448
5	H3K9(Ac) (1-20)	ARTKQTARK(Ac)STGGKAPRKQL	12.83	-1.610
6	H3K4(Me3)(1-21)	ARTK(Me3)QTARKSTGGKAPRKQLA	12.83	-1.448
7	H3K4(Me3)K9(Ac)S10(P) (1-21)	ARTK(Me3)QTARK(Ac)S(P)TGGKAPRKQLA	12.83	-1.448
8	H3K79(Me3),amide (69-89)	RLVREIAQDFK(Me3)TDLRFQSSAVK-NH2	9.98	-0.518
9	H3K79(Me3)T80(P),Biotin (69-89)	RLVREIAQDFK(Me3)T(P)DLRFQSSAVK(Biotin)	9.98	-0.518
10	H3K9(Me3) (3-17)	TKQTARK(Me3)STGGKAPR	12.02	-1.727
11	H3K4(Me3)(1-10)	ARTK(Me3)QTARKS	12.02	-1.890
12	H3K27(Me3) (23-34)	KAARK(Me3)SAPATGG	11.17	-0.750
13	H3K36(Me3)(31-41)	STGGVK(Me3)KPHRY	10.29	-1.500
14	H3K79(Me3) (73-83)	EIAQDFK(Me3)TDLR	4.56	-0.927

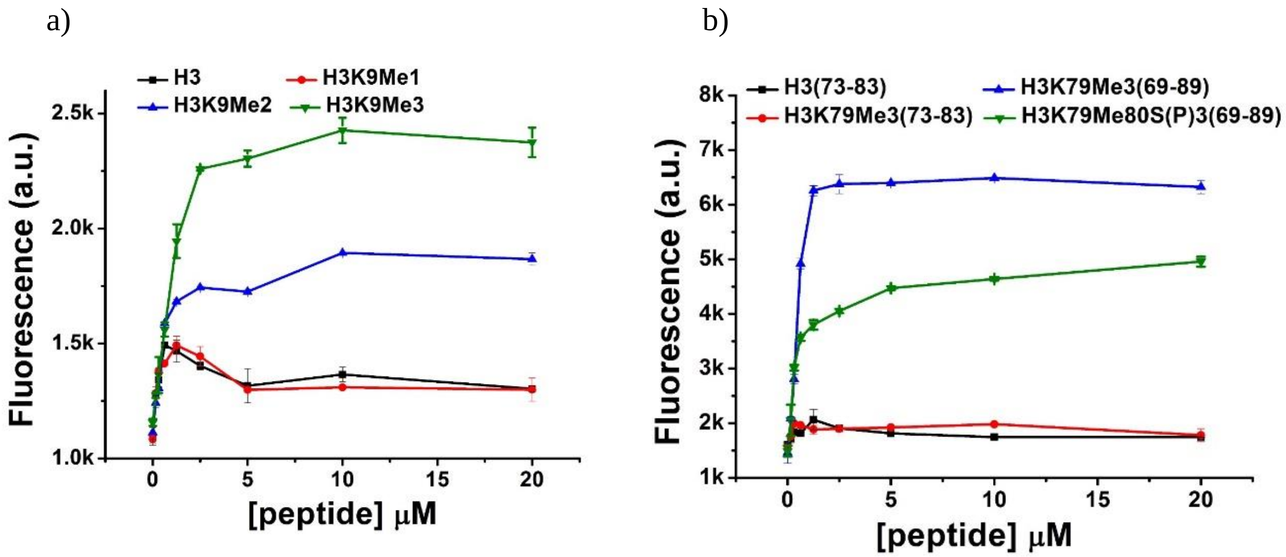

Figure S-25. Fluorescence recovery induced by mixing a) the H3K9 (1-21) peptide series with different methylation levels with the sensor of **1•4** (3 and 4 μM, respectively) at pH 9.0, and the H3K79 series with the sensor of **2•4** (5 and 4 μM, respectively) at pH 9.0. Peptide concentrations increased from 0 to 20 μM.

Table S4. Dissociation constants obtained by fitting the fluorescence recovery curves shown in Fig. S8 to the Hill equation as described in the Methods section.

Parameter	H3K79Me$_3$(69-89)	H3K79Me$_3$T80(P)(69-89)	H3K9Me$_2$(1-21)	K9Me$_3$(1-21)
k (μM)	0.50±0.04	0.42±1.39	0.41±1.00	0.96±0.18
n	3.08	0.62	0.77	2.27
R^2	0.9972	0.9783	0.9095	0.9924

4. References:

1. B. Soberats, E. Sanna, G. Martorell, C. Rotger and A. Costa, *Org Lett*. 2014, **16**, 2480-2483.
2. M. Obata, M. Morita, K. Nakase, K. Mitsuo, K. Asai, S. Hirohara and S. Yano, *J. Polym. Sci., Part A: Polym. Chem*, 2007, **45**, 2876-2885.
3. Y.-J. Ghang, L. Perez, M.A. Morgan, F. Si, O.M. Hamdy, C.N. Beecher, C.K. Larive, R.R. Julian, W. Zhong, Q. Cheng and R.J. Hooley, *Soft Matter* 2014**, 10**, 9651-9656.
